# Selection for resistance to oseltamivir in seasonal and pandemic H1N1 influenza and widespread co-circulation of the lineages

**DOI:** 10.1186/1476-072X-9-13

**Published:** 2010-02-24

**Authors:** Daniel A Janies, Igor O Voronkin, Jonathon Studer, Jori Hardman, Boyan B Alexandrov, Travis W Treseder, Chandni Valson

**Affiliations:** 1 Department of Biomedical Informatics, The Ohio State University, College of Medicine, Columbus, OH 43210 USA

## Abstract

**Background:**

In Spring 2009, a novel reassortant strain of H1N1 influenza A emerged as a lineage distinct from seasonal H1N1. On June 11, the World Heath Organization declared a pandemic - the first since 1968. There are currently two main branches of H1N1 circulating in humans, a seasonal branch and a pandemic branch. The primary treatment method for pandemic and seasonal H1N1 is the antiviral drug Tamiflu^® ^(oseltamivir). Although many seasonal H1N1 strains around the world are resistant to oseltamivir, initially, pandemic H1N1 strains have been susceptible to oseltamivir. As of February 3, 2010, there have been reports of resistance to oseltamivir in 225 cases of H1N1 pandemic influenza. The evolution of resistance to oseltamivir in pandemic H1N1 could be due to point mutations in the neuraminidase or a reassortment event between seasonal H1N1 and pandemic H1N1 viruses that provide a neuraminidase carrying an oseltamivir-resistant genotype to pandemic H1N1.

**Results:**

Using phylogenetic analysis of neuraminidase sequences, we show that both seasonal and pandemic lineages of H1N1 are evolving to direct selective pressure for resistance to oseltamivir. Moreover, seasonal lineages of H1N1 that are resistant to oseltamivir co-circulate with pandemic H1N1 throughout the globe. By combining phylogenetic and geographic data we have thus far identified 53 areas of co-circulation where reassortment can occur. At our website POINTMAP, http://pointmap.osu.edu we make available a visualization and an application for updating these results as more data are released.

**Conclusions:**

As oseltamivir is a keystone of preparedness and treatment for pandemic H1N1, the potential for resistance to oseltamivir is an ongoing concern. Reassortment and, more likely, point mutation have the potential to create a strain of pandemic H1N1 against which we have a reduced number of treatment options.

## Background

In Spring 2009, a novel reassortant strain of H1N1 influenza A emerged as a lineage distinct from seasonal H1N1. On June 11, the World Heath Organization declared a pandemic - the first since 1968 [1]. There are currently two main branches of H1N1 circulating in humans, a seasonal branch and a pandemic branch. The primary treatment for patients infected with influenza A is the antiviral drug Tamiflu^® ^(oseltamivir). Resistance to oseltamivir can occur due to a point mutation in any of several regions of the neuraminidase protein of the virus. Although many seasonal H1N1 viruses isolated around the world are resistant to oseltamivir [2,3], initially, most pandemic H1N1 isolates have been susceptible to oseltamivir. As of February 3, 2010, there have been reports of resistance to oseltamivir in 225 cases of H1N1 pandemic influenza [4]. Resistance to oseltamivir in pandemic H1N1 can present itself in non-exclusive patterns at various scales: 1) sporadic evolution within an infected patient in response to treatment [5], 2) evolution of resistance to oseltamivir in an infected patient infected and transfer of the strain among personal contacts [6] 3) maintenance of a genotype that confers resistance to oseltamivir in a viral lineage due to selection pressure [7] and or 4) a reassortment event between oseltamivir-resistant seasonal H1N1 and pandemic H1N1 viruses. This event could provide a neuraminidase segment that carries a genotype that confers oseltamivir resistance to pandemic H1N1 [7].

Resistance to oseltamivir in H1N1 can occur due to a point mutation at one of several sites in the neuraminidase (NA) protein (e.g., D79G, S247G or S247N, and H275Y) [8]. Resistance to Relenza^® ^(zanamivir) in H1N1 can occur due to point mutations including H126N or Q136K in NA [8,9]. We examined sequence diversity at key sites, selective pressure on NA codons, and geographic co-circulation among H1N1 lineages causing seasonal and pandemic influenza. Previous global surveys on seasonal H1N1 found low levels of resistance to oseltamivir in the first three years of their use up to August 31, 2002 [10]. We focused on high quality data for NA genetic sequence, geographic, and temporal information. We removed laboratory and host-adapted isolates, as well as isolates that were partially sequenced or caused mutations that broke the reading frame of the multiple sequence alignment. We included 1210 seasonal H1N1 NA segments isolated around the world between September 2004 and December 2009 (additional files 1 and 2). For pandemic H1N1, we included 1824 NA segments isolated between March 2009 and December 2009 (additional files 3 and 4). We developed a web application, called POINTMAP http://pointmap.osu.edu, to plot the place of isolation of viruses and to distribute our data and results. Our data reflect a non-overlapping set of sequences available in data repositories including: The National Institutes of Health's GenBank http://ncbi.nlm.nih.gov and The Global Initiative on Sharing of All Influenza Data (GISAID; http://www.gisaid.org).

## Results

The best likelihood scores were as follows: for the pandemic H1N1 dataset ln - 9857.691488 (additional file 5) and for the seasonal H1N1 dataset ln -13871.895684 (additional file 6).

To detect positive selection we used the criterion of statistically significant bias of non-synonymous mutations (dN) relative to synonymous mutations (dS) at a codon [11]. For the seasonal H1N1 lineage, we see evidence for positive natural selection for resistance to oseltamivir conferred by the genotype 275Y (dN-dS = 6.097; p = 0.035). The codon corresponding to amino acid position 275 is the sole region on the NA segment currently exhibiting significant dN-dS bias.

In the pandemic clade of H1N1, we see evidence for positive natural selection for resistance to oseltamivir as conferred by genotype 275Y (dN-dS = 7.69; p = 0.006). There is also significant dN-dS bias at codon 248 (dN-dS = 5.18; p = 0.031). Of the 225 reports of cases of resistance to oseltamivir among pandemic H1N1 [4], a subset of 28 isolates has been sequenced and put in public databases as of January 4, 2010. Based on our NA phylogeny, the members of the H1N1 pandemic lineage containing the 275Y NA genotype include: isolates from patients infected with pandemic H1N1 and treated with oseltamivir in the United States (Washington State) [5], two clades of isolates restricted to Japan (Yamaguchi plus Chiba; Shiga plus Niigata), a clade of isolates with a worldwide distribution (Japan, Denmark, plus the United States), and various unrelated isolates from China, Israel, Africa, the United States, Japan, and Europe. There are also reports of oseltamivir resistance in pandemic H1N1 isolates from Singapore but their NA has not yet been sequenced.

In the case of seasonal H1N1, some workers have dismissed the possibility of H1N1 responding to use of drugs based on low sales of oseltamivir in Norway between 2002 and 2007 [12]. However, large numbers of doses of oseltamivir have been purchased by many entities around the world. These doses have been recently used widely in treatment and prophylaxis of mild cases [13], especially in Japan [14]. Moreover, any illegal trade and use of drugs around the world will be difficult to measure. There are reports of evolution of resistance and spread among patients as a result of the use of oseltamivir in prophylaxis [5,6].

The mutations D79G, S247G, and S247N in NA also confer resistance to oseltamivir [8]. For amino acid positions 79 and 247 there is very little variation in seasonal H1N1. For amino acid position 79, most of the seasonal clade contains the wild type, 79D. The pandemic clade largely contains 79S. For position 247 in the seasonal clade, most isolates contain the wild type, 247S. Two seasonal isolates from humans in Montserrat contains 247N. Regarding position 247 in the pandemic clade, all isolates thus far sequenced and put into public databases contain the wild type, 247S.

The mutations H126N or Q136K in NA confer resistance to zanamivir in H1N1 [8,9]. For amino acid position 126 in NA, the seasonal clade contains the wild type, 126H. The pandemic clade contains largely 126P, which merits further study.

Due to the increasing importance of zanamivir, we provide a Keyhole Mark-up Language (KML) file in which we mapped isolates from Australia, Thailand, New Zealand, Brazil, Nicaragua, and Macau that contain the genotype 136K in NA for the seasonal clade (additional file 7). The pandemic clade uniformly contains the wild type, 136Q in NA.

**Figure 1:**
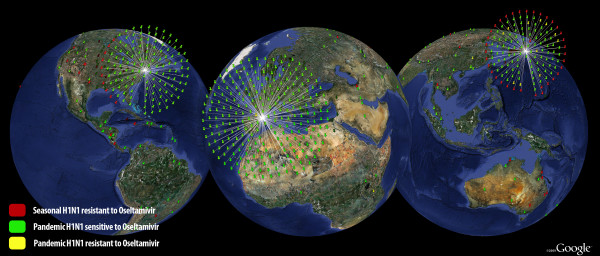
**Screen shot of an interactive visualization of populations of oseltamivir-resistant seasonal H1N1 influenza and pandemic H1N1 influenza in co-circulation across the globe**. The visualization is available via a web browser on our website POINTMAP http://pointmap.osu.edu or in additional file 8 associated with the paper.

As either selection or reassortment can lead to strains of pandemic H1N1 that are resistant to drugs, we complement analyses of selection with a map of co-occurrence of oseltamivir-resistant seasonal H1N1 and pandemic H1N1 (Figure 1). We found 53 regions in which these influenza strains co-circulate (Table 1). A subset of six of these 53 regions contains pandemic H1N1 isolates with NA segments that carry the genotype 275Y in NA. This subset includes regions within Japan and the United States. To precisely illustrate the 53 areas of co-circulation of seasonal and pandemic H1N1 we distribute a KML file (see http://pointmap.osu.edu or additional file 8).

**Table 1:** Areas in which populations of pandemic H1N1 and oseltamivir-resistant seasonal H1N1 and co-circulate.

City	State or Region	Country
		China
		Honduras
	Aichi-ken	Japan
	Akita-ken	Japan
	Gifu-ken	Japan
	Kagoshima-ken	Japan
	Niigata-ken	Japan
	Wakayama-ken	Japan
Yokohama-shi		Japan
		Kenya
		Mexico
		Myanmar
		Puerto Rico
St. Petersburg		Russia
		Singapore
		South Korea
		Taiwan
		Thailand
	Alaska	United States
	Arizona	United States
	California	United States
	Colorado	United States
	Delaware	United States
	Florida	United States
	Hawaii	United States
	Illinois	United States
	Indiana	United States
	Iowa	United States
	Kentucky	United States
	Maryland	United States
	Massachusetts	United States
	Michigan	United States
	Minnesota	United States
St. Louis	Missouri	United States
	Montana	United States
	Nebraska	United States
	Nevada	United States
	New Mexico	United States
Steuben County	New York	United States
Ulster County	New York	United States
	North Dakota	United States
	Ohio	United States
	Oregon	United States
	Pennsylvania	United States
	Rhode Island	United States
	South Dakota	United States
	Tennessee	United States
	Utah	United States
	Vermont	United States
	Washington	United States
	West Virginia	United States
	Wisconsin	United States
	Wyoming	United States

## Discussion

Here we demonstrate positive selective pressure for resistance to oseltamivir conferred by genotype 275Y in NA in both seasonal H1N1 and pandemic H1N1. This result shows the importance of judicious use of antiviral medication. H275Y mutations in seasonal H1N1 began to appear in 2007. A lineage of seasonal H1N1 became fixed for the genotype 275Y in Europe in 2008 and spread across the globe. H275Y mutations began to appear in clades within the pandemic lineage of H1N1 in June 2009. Reports of resistance cases have steadily increased through 2009 [4] and more sequence data is expected.

As seasonal H1N1 is an older lineage, we expect selection for resistance to oseltamivir and subsequent fixation of 275Y in seasonal H1N1 lineages to foreshadow the evolutionary trajectory of pandemic H1N1. Based on sequence data available to date, pandemic H1N1 is not yet fixed for 275Y in NA. However, there is enough non-synonymous mutation in pandemic H1N1 to measure positive selection with statistical significance.

Codon 275 is the only position exhibiting dN-dS bias in the NA segment of seasonal H1N1. This contradicts the argument that codon 275 is not changing due to direct selection but rather is genetically linked ("hitch-hiking") to another position that is associated with a phenotype other than drug-resistance that is under positive selection [15]. In the NA segment of pandemic H1N1, there are two sites exhibiting dN-dS bias: codon 275 and codon 248. Thus we examined the possibility of genetic linkage within NA. In doing so, we can also refute the hitch-hiking argument for the evolution of NA in pandemic H1N1. Under genetic linkage, one would expect co-variation between H275Y and N248D. However in contrast, the mutations N248D and H275Y have very different phylogenetic distributions. The genotype 275Y, as described above, occurs in 28 isolates in a few clades and multiple unrelated lineages. The N248D mutation occurs in a handful of isolates from the origins of the pandemic, but in later isolates the 248D genotype becomes fixed in an immense clade of over 1000 isolates. A reversal mutation (D248N) occurs in another large clade at the crown of the tree. The N248D mutation is reported to alter an antibody recognition site and thus has implications for vaccine development [16]. Phenotypes associated with this polymorphism merit further study.

In addition, a reassortment event in a co-infected host could lead to emergence of a drug-resistant strain of epidemic H1N1. Our map illustrates that there is abundant opportunity for co-infection and reassortment of a NA segment from oseltamivir resistant seasonal H1N1 ancestors to pandemic H1N1 descendants. There are reports of mixed infections of pandemic H1N1 and seasonal influenza viruses in China [GenBank: CY048942]. This lends support to the hypothesis that reassortment has the potential for the evolution of drug resistance and merits further study.

Based on limited testing, there are reports that pandemic H1N1 is becoming dominant in the Western hemisphere, Japan, and Europe. However in China, seasonal strains remain in co-circulation with pandemic H1N1 [17]. If seasonal H1N1 becomes infrequent, the possibility of reassortment diminishes, as co-infections will be rare. However the short-term lull in seasonal H1N1 activity may not signal its complete extinction. A study posted as a knol (unit of knowledge) on co-infection in ferrets that found no reassortment [18]. If ferret studies model the behavior of the viruses in human cells, this result conflicts with the hypothesis that reassortment will occur between pandemic and seasonal H1N1.

Mathematical models have suggested that during a pandemic, treatment with more than one drug may be an effective "hedging" strategy to abate widespread drug resistance among the population while still treating patients [19]. These models have been challenged by others who are skeptical about the presence of direct selection on the drug target [20]. Here we demonstrate direct selection for resistance to oseltamivir in both seasonal and pandemic lineages of H1N1. We also show that resistance to zanamivir is currently low, thus use of this drug is currently a sound alternative to oseltamivir.

## Conclusions

Judicious prescription of antivirals is important in order to maintain the ability to use antivirals to treat high-risk patients. Furthermore, precise diagnosis of influenza A informed by point-of-care genotyping of the pathogen can help target the proper antiviral to each patient. This study illustrates how genomics technology, bioinformatics, and geographic information systems can be of immediate applicability to personalize treatment of infectious diseases. In addition to data collection, analyses are needed to turn raw data into prospective public health intelligence on drug resistance in a regionally specific and easy to visualize manner.

## Methods

We collected sequence data from GenBank http://www.ncbi.nlm.nih.gov and GISAID http://www.gisaid.org. Geographic and temporal information was extracted from the 'TSeq_orgname' field in GenBank's TinySeq XML records or from the FASTA label of a GISAID record.

We converted place names to latitude and longitude in decimal degrees corresponding to the centroid of the place based on queries to geonames http://geonames.org. We performed multiple sequence alignments on nucleotide sequences using default parameters with CLUSTALW-MPI (version 0.13) [21]. We then ran MODELTEST version 3.8 [22] as implemented in PAUP version 4.0b10 [23] to choose best-fit model models of nucleotide substitution based on the Akaike Information Criterion not using branch lengths as parameters. As suggested by MODELTEST, phylogenetic analyses were for both datasets were conducted under the GTRGAMMAI model of nucleotide substitution. Both datasets were run for 100 replicates with RAXML-HPC-MPI (version 4.0.4) [24]. Analyses of selection were performed using single likelihood ancestor counting (SLAC) with HYPHY (version 0.9920070619 beta) [11] to measure synonymous and non-synonymous substitution for each codon at the p < = 0.05 level for statistical significance. For analyses of selection we used all isolates in each dataset.

We used the following criteria for analysis of co-circulation. We selected only the clade of the seasonal lineage in which the genotype 275Y (conferring resistance to oseltamivir) is fixed. We reduced the oseltamivir-resistant fixed seasonal clade further by using isolates from 2009. We compared latitude and longitude data from the 2009 oseltamivir-resistant fixed seasonal set to all latitude and longitude data for the pandemic lineage except for the outgroup.

The outgroup for seasonal data was [GISAID: EPI182496] A/Berlin/6/2006 and for pandemic data was [GISAID: EPI161647] A/swine/Italy/247578/2004. Outgroups were discovered based on searches of larger datasets spanning back to 1918.

## Competing interests

The authors declare that they have no competing interests.

## Authors' contributions

DJ performed analyses and wrote the manuscript with editing by JS and IV. IV and DJ developed specialized scripts for the work. JH, BA, TT, and IV developed the POINTMAP application and executed analyses. IV, JS and CV collected data, and BA created the figure and KML files. All authors have read and approve of the final version of the paper.

## Supplementary Material

Additional file 1 **Character optimization of H275Y on a tree based on NA segments from the pandemic H1N1 lineage**. This file is in scalable portable document format. The mutation H275Y visualized in color (green = H = susceptible to oseltamivir: red = Y = resistant to oseltamivir) on the best tree. Click here for file

Additional file 2 **Accession numbers of the neuraminidase nucleotide sequences used in the phylogenetic and geographic study of pandemic H1N1 influenza A**. GISAID sequences are available at http://www.gisaid.org. GenBank sequences are available at http://ncbi.nlm.nih.gov. Click here for file

Additional file 3 **Character optimization of H275Y on a tree based on NA segments from the seasonal H1N1 lineage**. This file is in scalable portable document format. The mutation H275Y visualized in color (green = H = susceptible to oseltamivir: red = Y = resistant to oseltamivir) on the best tree. Click here for file

Additional file 4 **Accession numbers of the neuraminidase nucleotide sequences used in the phylogenetic and geographic study of seasonal H1N1 influenza A**. GISAID sequences are available at http://www.gisaid.org. GenBank sequences are available at http://ncbi.nlm.nih.gov. Click here for file

Additional file 5 **Heuristic maximum likelihood tree based on NA segments from the pandemic H1N1 lineage**. The file is in nexus format. Click here for file

Additional file 6 **Heuristic maximum likelihood tree based on NA segments from the seasonal H1N1 lineage**. The file is in nexus format. Click here for file

Additional file 7 **An interactive visualization of populations of zanamivir-resistant seasonal H1N1**. A KML file suitable for viewing with Google Earth™ http://earth.google.com or other virtual globe software. Once the user opens the file in Google Earth, the user will see white points that represent isolates of seasonal H1N1 that are resistant to zanamivir. Click here for file

Additional file 8 **An interactive visualization of populations of oseltamivir-resistant seasonal H1N1 influenza and pandemic influenza in co-circulation across the globe**. A KML file suitable for viewing with Google Earth™ http://earth.google.com or other virtual globe software. Once the user opens the KML file in Google Earth, the user will see red points that represent isolates of seasonal H1N1 that are resistant to oseltamivir. Green points represent isolates of pandemic H1N1 that are susceptible to oseltamivir. Yellow points represent isolates of pandemic H1N1 that are resistant to oseltamivir. Clicking on a point will reveal if various isolates co-circulate in that region. Click here for file

Additional file 9 **Acknowledgements**. Acknowledgements for researchers and institutions who submitted sequence data to GISAID and GenBank. Click here for file
