# Supplementary figures and images for: Selection for resistance to oseltamivir in seasonal and pandemic H1N1 influenza and widespread co-circulation of the lineages
Source: Int J Health Geogr. 2010 Feb 24;9:13. doi: 10.1186/1476-072X-9-13 (PMC2882220; doi:10.1186/1476-072X-9-13)

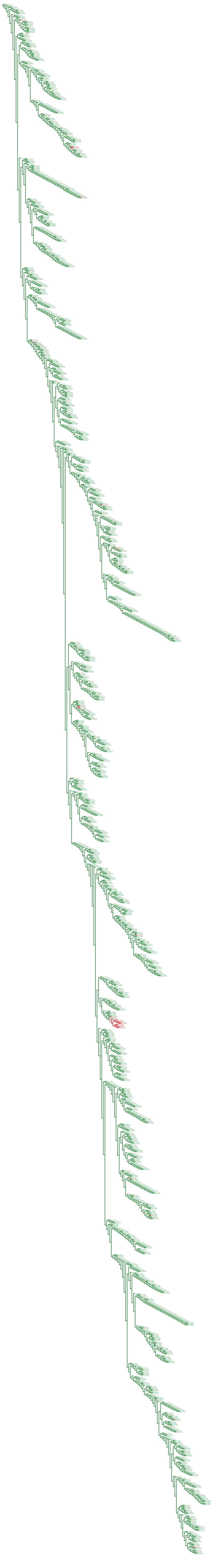

Supplement: Additional file 1 — Character optimization of H275Y on a tree based on NA segments from the pandemic H1N1 lineage. This file is in scalable portable document format. The mutation H275Y visualized in color (green = H = susceptible to oseltamivir: red = Y = resistant to oseltamivir) on the best tree. [file 1476-072X-9-13-S1.PDF]

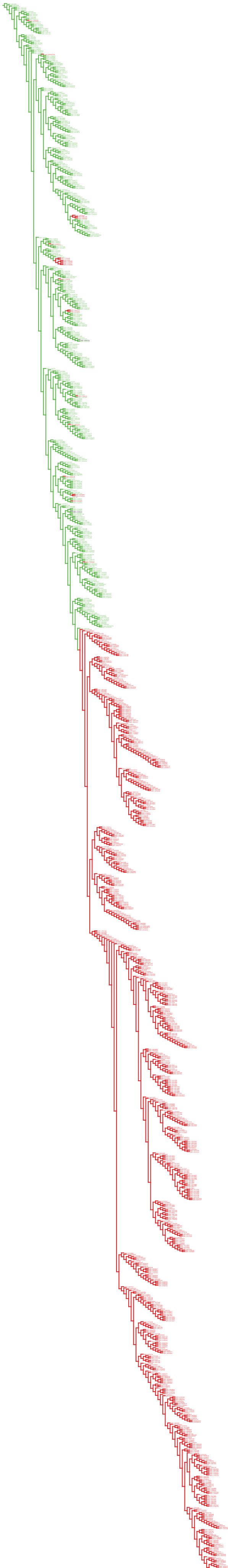

Supplement: Additional file 3 — Character optimization of H275Y on a tree based on NA segments from the seasonal H1N1 lineage. This file is in scalable portable document format. The mutation H275Y visualized in color (green = H = susceptible to oseltamivir: red = Y = resistant to oseltamivir) on the best tree. [file 1476-072X-9-13-S3.PDF]
