# Supplementary material for: Selection for resistance to oseltamivir in seasonal and pandemic H1N1 influenza and widespread co-circulation of the lineages
Source: Int J Health Geogr. 2010 Feb 24;9:13. doi: 10.1186/1476-072X-9-13 (PMC2882220; doi:10.1186/1476-072X-9-13)
Supplement: Additional file 2 — Accession numbers of the neuraminidase nucleotide sequences used in the phylogenetic and geographic study of pandemic H1N1 influenza A. GISAID sequences are available at http://www.gisaid.org. GenBank sequences are available at http://ncbi.nlm.nih.gov. [file 1476-072X-9-13-S2.DOC]

| GISAID | GenBank |
| --- | --- |
| EPI161647 | CY052027 |
| EPI176472 |  |
| EPI177278 |  |
| EPI177280 |  |
| EPI177316 |  |
| EPI177366 |  |
| EPI177932 |  |
| EPI177949 |  |
| EPI178255 |  |
| EPI178260 |  |
| EPI178265 |  |
| EPI178270 |  |
| EPI178286 |  |
| EPI178287 |  |
| EPI178288 |  |
| EPI178417 |  |
| EPI178430 |  |
| EPI178440 |  |
| EPI178446 |  |
| EPI178470 |  |
| EPI178474 |  |
| EPI178477 |  |
| EPI178503 |  |
| EPI178509 |  |
| EPI178516 |  |
| EPI178518 |  |
| EPI178939 |  |
| EPI178940 |  |
| EPI178986 |  |
| EPI179010 |  |
| EPI179011 |  |
| EPI179012 |  |
| EPI179013 |  |
| EPI179014 |  |
| EPI179015 |  |
| EPI179041 |  |
| EPI179042 |  |
| EPI179072 |  |
| EPI179081 |  |
| EPI179084 |  |
| EPI179088 |  |
| EPI179090 |  |
| EPI179095 |  |
| EPI179101 |  |
| EPI179103 |  |
| EPI179115 |  |
| EPI179122 |  |
| EPI179145 |  |
| EPI179176 |  |
| EPI179184 |  |
| EPI179192 |  |
| EPI179202 |  |
| EPI179306 |  |
| EPI179362 |  |
| EPI179374 |  |
| EPI179386 |  |
| EPI179392 |  |
| EPI179408 |  |
| EPI179424 |  |
| EPI179430 |  |
| EPI179466 |  |
| EPI179467 |  |
| EPI179468 |  |
| EPI179469 |  |
| EPI179470 |  |
| EPI179481 |  |
| EPI179493 |  |
| EPI179584 |  |
| EPI179586 |  |
| EPI179588 |  |
| EPI179590 |  |
| EPI180014 |  |
| EPI180015 |  |
| EPI180016 |  |
| EPI180017 |  |
| EPI180018 |  |
| EPI180019 |  |
| EPI180020 |  |
| EPI180021 |  |
| EPI180022 |  |
| EPI180024 |  |
| EPI180044 |  |
| EPI180051 |  |
| EPI180055 |  |
| EPI180061 |  |
| EPI180068 |  |
| EPI180215 |  |
| EPI180218 |  |
| EPI180225 |  |
| EPI180226 |  |
| EPI180227 |  |
| EPI180228 |  |
| EPI180229 |  |
| EPI180236 |  |
| EPI180259 |  |
| EPI180271 |  |
| EPI180342 |  |
| EPI180364 |  |
| EPI180372 |  |
| EPI180380 |  |
| EPI180388 |  |
| EPI180404 |  |
| EPI180412 |  |
| EPI180444 |  |
| EPI180452 |  |
| EPI180460 |  |
| EPI180492 |  |
| EPI180500 |  |
| EPI180508 |  |
| EPI180516 |  |
| EPI180524 |  |
| EPI180540 |  |
| EPI180555 |  |
| EPI180558 |  |
| EPI180564 |  |
| EPI180570 |  |
| EPI180576 |  |
| EPI180603 |  |
| EPI180637 |  |
| EPI180701 |  |
| EPI180705 |  |
| EPI180710 |  |
| EPI180730 |  |
| EPI180732 |  |
| EPI180733 |  |
| EPI180734 |  |
| EPI180741 |  |
| EPI180749 |  |
| EPI180757 |  |
| EPI180760 |  |
| EPI180965 |  |
| EPI180973 |  |
| EPI180981 |  |
| EPI180989 |  |
| EPI180997 |  |
| EPI181005 |  |
| EPI181013 |  |
| EPI181021 |  |
| EPI181028 |  |
| EPI181032 |  |
| EPI181045 |  |
| EPI181052 |  |
| EPI181059 |  |
| EPI181062 |  |
| EPI181070 |  |
| EPI181078 |  |
| EPI181086 |  |
| EPI181094 |  |
| EPI181102 |  |
| EPI181119 |  |
| EPI181127 |  |
| EPI181135 |  |
| EPI181145 |  |
| EPI181153 |  |
| EPI181161 |  |
| EPI181169 |  |
| EPI181277 |  |
| EPI181320 |  |
| EPI181328 |  |
| EPI181336 |  |
| EPI181352 |  |
| EPI181356 |  |
| EPI181671 |  |
| EPI181679 |  |
| EPI181687 |  |
| EPI181695 |  |
| EPI181703 |  |
| EPI181711 |  |
| EPI181719 |  |
| EPI181727 |  |
| EPI181746 |  |
| EPI181844 |  |
| EPI181862 |  |
| EPI181864 |  |
| EPI181866 |  |
| EPI181868 |  |
| EPI181870 |  |
| EPI181874 |  |
| EPI181901 |  |
| EPI182198 |  |
| EPI182206 |  |
| EPI182211 |  |
| EPI182219 |  |
| EPI182227 |  |
| EPI182235 |  |
| EPI182243 |  |
| EPI182251 |  |
| EPI182259 |  |
| EPI182269 |  |
| EPI182392 |  |
| EPI182417 |  |
| EPI182424 |  |
| EPI182550 |  |
| EPI182553 |  |
| EPI182562 |  |
| EPI182601 |  |
| EPI182676 |  |
| EPI182684 |  |
| EPI182691 |  |
| EPI182699 |  |
| EPI182707 |  |
| EPI182715 |  |
| EPI182723 |  |
| EPI182731 |  |
| EPI182739 |  |
| EPI182747 |  |
| EPI182755 |  |
| EPI182763 |  |
| EPI182771 |  |
| EPI182779 |  |
| EPI182787 |  |
| EPI182795 |  |
| EPI182861 |  |
| EPI182862 |  |
| EPI182864 |  |
| EPI182866 |  |
| EPI182906 |  |
| EPI182978 |  |
| EPI182979 |  |
| EPI182980 |  |
| EPI182981 |  |
| EPI182982 |  |
| EPI182983 |  |
| EPI182984 |  |
| EPI182985 |  |
| EPI182993 |  |
| EPI182998 |  |
| EPI183004 |  |
| EPI183012 |  |
| EPI183014 |  |
| EPI183016 |  |
| EPI183018 |  |
| EPI183020 |  |
| EPI183062 |  |
| EPI183097 |  |
| EPI183098 |  |
| EPI183126 |  |
| EPI183136 |  |
| EPI183141 |  |
| EPI183146 |  |
| EPI183148 |  |
| EPI183159 |  |
| EPI183164 |  |
| EPI183180 |  |
| EPI183208 |  |
| EPI183215 |  |
| EPI183235 |  |
| EPI183239 |  |
| EPI183252 |  |
| EPI183257 |  |
| EPI183266 |  |
| EPI183279 |  |
| EPI183318 |  |
| EPI183360 |  |
| EPI183367 |  |
| EPI183372 |  |
| EPI183378 |  |
| EPI183395 |  |
| EPI183477 |  |
| EPI183478 |  |
| EPI183479 |  |
| EPI183480 |  |
| EPI183481 |  |
| EPI183482 |  |
| EPI183483 |  |
| EPI183484 |  |
| EPI183485 |  |
| EPI183486 |  |
| EPI183487 |  |
| EPI183488 |  |
| EPI183489 |  |
| EPI183490 |  |
| EPI183491 |  |
| EPI183492 |  |
| EPI183493 |  |
| EPI183494 |  |
| EPI183495 |  |
| EPI183496 |  |
| EPI183497 |  |
| EPI183498 |  |
| EPI183499 |  |
| EPI183500 |  |
| EPI183501 |  |
| EPI183502 |  |
| EPI183503 |  |
| EPI183504 |  |
| EPI183505 |  |
| EPI183506 |  |
| EPI183507 |  |
| EPI183508 |  |
| EPI183509 |  |
| EPI183510 |  |
| EPI183511 |  |
| EPI183512 |  |
| EPI183513 |  |
| EPI183514 |  |
| EPI183515 |  |
| EPI183516 |  |
| EPI183517 |  |
| EPI183518 |  |
| EPI183519 |  |
| EPI183520 |  |
| EPI183521 |  |
| EPI183522 |  |
| EPI183523 |  |
| EPI183524 |  |
| EPI183525 |  |
| EPI183526 |  |
| EPI183527 |  |
| EPI183528 |  |
| EPI183529 |  |
| EPI183530 |  |
| EPI183531 |  |
| EPI183532 |  |
| EPI183533 |  |
| EPI183534 |  |
| EPI183535 |  |
| EPI183536 |  |
| EPI183537 |  |
| EPI183538 |  |
| EPI183539 |  |
| EPI183644 |  |
| EPI183672 |  |
| EPI183673 |  |
| EPI183674 |  |
| EPI183675 |  |
| EPI183680 |  |
| EPI183685 |  |
| EPI183731 |  |
| EPI183740 |  |
| EPI183748 |  |
| EPI183772 |  |
| EPI183788 |  |
| EPI183804 |  |
| EPI183812 |  |
| EPI183820 |  |
| EPI183828 |  |
| EPI183836 |  |
| EPI183847 |  |
| EPI183848 |  |
| EPI184069 |  |
| EPI184223 |  |
| EPI184231 |  |
| EPI184248 |  |
| EPI184285 |  |
| EPI184377 |  |
| EPI184597 |  |
| EPI184647 |  |
| EPI184658 |  |
| EPI184665 |  |
| EPI184675 |  |
| EPI184687 |  |
| EPI184692 |  |
| EPI184697 |  |
| EPI184701 |  |
| EPI184705 |  |
| EPI184711 |  |
| EPI184715 |  |
| EPI184718 |  |
| EPI184722 |  |
| EPI184725 |  |
| EPI184730 |  |
| EPI184734 |  |
| EPI184737 |  |
| EPI184739 |  |
| EPI184741 |  |
| EPI184748 |  |
| EPI184753 |  |
| EPI184785 |  |
| EPI184786 |  |
| EPI184787 |  |
| EPI184788 |  |
| EPI184789 |  |
| EPI184790 |  |
| EPI184791 |  |
| EPI184792 |  |
| EPI184851 |  |
| EPI184854 |  |
| EPI184857 |  |
| EPI184862 |  |
| EPI184867 |  |
| EPI184869 |  |
| EPI184871 |  |
| EPI184873 |  |
| EPI184876 |  |
| EPI184878 |  |
| EPI184881 |  |
| EPI184884 |  |
| EPI184902 |  |
| EPI184908 |  |
| EPI184919 |  |
| EPI184928 |  |
| EPI184936 |  |
| EPI184943 |  |
| EPI184946 |  |
| EPI184954 |  |
| EPI184961 |  |
| EPI184964 |  |
| EPI184973 |  |
| EPI184978 |  |
| EPI185058 |  |
| EPI185065 |  |
| EPI185166 |  |
| EPI185185 |  |
| EPI185188 |  |
| EPI185190 |  |
| EPI185192 |  |
| EPI185195 |  |
| EPI185198 |  |
| EPI185201 |  |
| EPI185203 |  |
| EPI185206 |  |
| EPI185208 |  |
| EPI185211 |  |
| EPI185214 |  |
| EPI185216 |  |
| EPI185224 |  |
| EPI185309 |  |
| EPI185589 |  |
| EPI185590 |  |
| EPI185596 |  |
| EPI185957 |  |
| EPI185958 |  |
| EPI185959 |  |
| EPI185960 |  |
| EPI185961 |  |
| EPI185962 |  |
| EPI185963 |  |
| EPI185964 |  |
| EPI185965 |  |
| EPI185966 |  |
| EPI185967 |  |
| EPI185968 |  |
| EPI185969 |  |
| EPI185970 |  |
| EPI185971 |  |
| EPI185972 |  |
| EPI185973 |  |
| EPI185974 |  |
| EPI185975 |  |
| EPI185976 |  |
| EPI185977 |  |
| EPI185978 |  |
| EPI185979 |  |
| EPI185980 |  |
| EPI185981 |  |
| EPI185982 |  |
| EPI185983 |  |
| EPI185984 |  |
| EPI185985 |  |
| EPI185986 |  |
| EPI185987 |  |
| EPI185988 |  |
| EPI185989 |  |
| EPI185990 |  |
| EPI185991 |  |
| EPI185992 |  |
| EPI185993 |  |
| EPI185994 |  |
| EPI186130 |  |
| EPI186232 |  |
| EPI186240 |  |
| EPI186276 |  |
| EPI186277 |  |
| EPI186280 |  |
| EPI186325 |  |
| EPI186595 |  |
| EPI186596 |  |
| EPI186597 |  |
| EPI186598 |  |
| EPI186599 |  |
| EPI186600 |  |
| EPI186601 |  |
| EPI186602 |  |
| EPI186603 |  |
| EPI186604 |  |
| EPI186605 |  |
| EPI186606 |  |
| EPI186607 |  |
| EPI186635 |  |
| EPI186643 |  |
| EPI186651 |  |
| EPI186659 |  |
| EPI186667 |  |
| EPI186675 |  |
| EPI186738 |  |
| EPI186739 |  |
| EPI186740 |  |
| EPI186741 |  |
| EPI186742 |  |
| EPI186743 |  |
| EPI186744 |  |
| EPI186745 |  |
| EPI186746 |  |
| EPI186747 |  |
| EPI186748 |  |
| EPI186749 |  |
| EPI186750 |  |
| EPI186751 |  |
| EPI186752 |  |
| EPI186753 |  |
| EPI186754 |  |
| EPI186755 |  |
| EPI186756 |  |
| EPI186757 |  |
| EPI186840 |  |
| EPI186846 |  |
| EPI187707 |  |
| EPI187709 |  |
| EPI187719 |  |
| EPI187727 |  |
| EPI187735 |  |
| EPI187743 |  |
| EPI187751 |  |
| EPI187767 |  |
| EPI187775 |  |
| EPI187783 |  |
| EPI188889 |  |
| EPI188913 |  |
| EPI188921 |  |
| EPI188929 |  |
| EPI188937 |  |
| EPI188945 |  |
| EPI188953 |  |
| EPI188969 |  |
| EPI188977 |  |
| EPI188985 |  |
| EPI188993 |  |
| EPI189001 |  |
| EPI189070 |  |
| EPI189146 |  |
| EPI189183 |  |
| EPI189190 |  |
| EPI189202 |  |
| EPI189207 |  |
| EPI189210 |  |
| EPI190200 |  |
| EPI190208 |  |
| EPI190216 |  |
| EPI190222 |  |
| EPI190446 |  |
| EPI190454 |  |
| EPI190462 |  |
| EPI190470 |  |
| EPI190478 |  |
| EPI190486 |  |
| EPI190494 |  |
| EPI190510 |  |
| EPI190518 |  |
| EPI190526 |  |
| EPI190534 |  |
| EPI190546 |  |
| EPI190633 |  |
| EPI190634 |  |
| EPI190769 |  |
| EPI190785 |  |
| EPI190793 |  |
| EPI190801 |  |
| EPI190809 |  |
| EPI190817 |  |
| EPI190825 |  |
| EPI190833 |  |
| EPI190841 |  |
| EPI190849 |  |
| EPI190857 |  |
| EPI190865 |  |
| EPI190873 |  |
| EPI190881 |  |
| EPI190889 |  |
| EPI190935 |  |
| EPI190936 |  |
| EPI190937 |  |
| EPI190938 |  |
| EPI190939 |  |
| EPI190940 |  |
| EPI190941 |  |
| EPI190942 |  |
| EPI190943 |  |
| EPI190944 |  |
| EPI190945 |  |
| EPI190946 |  |
| EPI190995 |  |
| EPI190996 |  |
| EPI190997 |  |
| EPI190998 |  |
| EPI190999 |  |
| EPI191000 |  |
| EPI191001 |  |
| EPI191002 |  |
| EPI191003 |  |
| EPI191004 |  |
| EPI191005 |  |
| EPI191006 |  |
| EPI191007 |  |
| EPI191008 |  |
| EPI191009 |  |
| EPI191010 |  |
| EPI191012 |  |
| EPI191013 |  |
| EPI191014 |  |
| EPI191015 |  |
| EPI191016 |  |
| EPI191017 |  |
| EPI191018 |  |
| EPI191019 |  |
| EPI191020 |  |
| EPI191773 |  |
| EPI191778 |  |
| EPI191936 |  |
| EPI191938 |  |
| EPI191960 |  |
| EPI191973 |  |
| EPI191979 |  |
| EPI191990 |  |
| EPI191995 |  |
| EPI192682 |  |
| EPI192715 |  |
| EPI192720 |  |
| EPI192747 |  |
| EPI192752 |  |
| EPI192760 |  |
| EPI192768 |  |
| EPI192784 |  |
| EPI192792 |  |
| EPI192800 |  |
| EPI192808 |  |
| EPI192816 |  |
| EPI192832 |  |
| EPI192840 |  |
| EPI192848 |  |
| EPI192856 |  |
| EPI192864 |  |
| EPI192872 |  |
| EPI192880 |  |
| EPI192888 |  |
| EPI192920 |  |
| EPI192928 |  |
| EPI192936 |  |
| EPI192944 |  |
| EPI192952 |  |
| EPI192960 |  |
| EPI192968 |  |
| EPI192984 |  |
| EPI193094 |  |
| EPI193159 |  |
| EPI193167 |  |
| EPI193175 |  |
| EPI193183 |  |
| EPI193191 |  |
| EPI193199 |  |
| EPI193207 |  |
| EPI193223 |  |
| EPI193231 |  |
| EPI193466 |  |
| EPI193474 |  |
| EPI193482 |  |
| EPI193495 |  |
| EPI193854 |  |
| EPI194069 |  |
| EPI194077 |  |
| EPI194085 |  |
| EPI194093 |  |
| EPI194101 |  |
| EPI194109 |  |
| EPI194130 |  |
| EPI194132 |  |
| EPI194134 |  |
| EPI194136 |  |
| EPI194138 |  |
| EPI194139 |  |
| EPI194143 |  |
| EPI194145 |  |
| EPI194149 |  |
| EPI194153 |  |
| EPI194159 |  |
| EPI194161 |  |
| EPI194178 |  |
| EPI194188 |  |
| EPI194191 |  |
| EPI194195 |  |
| EPI194200 |  |
| EPI194201 |  |
| EPI194204 |  |
| EPI194206 |  |
| EPI194208 |  |
| EPI194211 |  |
| EPI194213 |  |
| EPI194216 |  |
| EPI194218 |  |
| EPI194219 |  |
| EPI194221 |  |
| EPI194222 |  |
| EPI194224 |  |
| EPI194228 |  |
| EPI194230 |  |
| EPI194237 |  |
| EPI194260 |  |
| EPI194261 |  |
| EPI194264 |  |
| EPI194275 |  |
| EPI194279 |  |
| EPI194281 |  |
| EPI194282 |  |
| EPI194291 |  |
| EPI194292 |  |
| EPI194294 |  |
| EPI194295 |  |
| EPI194297 |  |
| EPI194318 |  |
| EPI194319 |  |
| EPI194320 |  |
| EPI194321 |  |
| EPI194335 |  |
| EPI194343 |  |
| EPI194359 |  |
| EPI194367 |  |
| EPI194375 |  |
| EPI194383 |  |
| EPI194391 |  |
| EPI194399 |  |
| EPI194407 |  |
| EPI194415 |  |
| EPI194423 |  |
| EPI194431 |  |
| EPI194439 |  |
| EPI194447 |  |
| EPI194455 |  |
| EPI194463 |  |
| EPI194471 |  |
| EPI194479 |  |
| EPI194487 |  |
| EPI194495 |  |
| EPI194503 |  |
| EPI194511 |  |
| EPI194519 |  |
| EPI194527 |  |
| EPI194535 |  |
| EPI194543 |  |
| EPI194551 |  |
| EPI194559 |  |
| EPI194567 |  |
| EPI194575 |  |
| EPI194583 |  |
| EPI194591 |  |
| EPI194599 |  |
| EPI194607 |  |
| EPI194615 |  |
| EPI194623 |  |
| EPI194631 |  |
| EPI194639 |  |
| EPI194647 |  |
| EPI194655 |  |
| EPI194663 |  |
| EPI194671 |  |
| EPI194679 |  |
| EPI194687 |  |
| EPI194695 |  |
| EPI194703 |  |
| EPI194711 |  |
| EPI194719 |  |
| EPI194727 |  |
| EPI194735 |  |
| EPI194743 |  |
| EPI194751 |  |
| EPI194759 |  |
| EPI194767 |  |
| EPI194775 |  |
| EPI194783 |  |
| EPI194791 |  |
| EPI194799 |  |
| EPI194807 |  |
| EPI194815 |  |
| EPI194823 |  |
| EPI194831 |  |
| EPI194839 |  |
| EPI194847 |  |
| EPI194855 |  |
| EPI194863 |  |
| EPI194871 |  |
| EPI194879 |  |
| EPI194895 |  |
| EPI194903 |  |
| EPI194911 |  |
| EPI194919 |  |
| EPI194927 |  |
| EPI194935 |  |
| EPI194943 |  |
| EPI194951 |  |
| EPI194959 |  |
| EPI194967 |  |
| EPI194975 |  |
| EPI194983 |  |
| EPI194991 |  |
| EPI194999 |  |
| EPI195007 |  |
| EPI195015 |  |
| EPI195023 |  |
| EPI195031 |  |
| EPI195039 |  |
| EPI195047 |  |
| EPI195055 |  |
| EPI195063 |  |
| EPI195071 |  |
| EPI195079 |  |
| EPI195087 |  |
| EPI195238 |  |
| EPI195279 |  |
| EPI195287 |  |
| EPI195295 |  |
| EPI195559 |  |
| EPI195567 |  |
| EPI195575 |  |
| EPI195579 |  |
| EPI195582 |  |
| EPI195585 |  |
| EPI195635 |  |
| EPI195637 |  |
| EPI195639 |  |
| EPI195641 |  |
| EPI195643 |  |
| EPI195645 |  |
| EPI195647 |  |
| EPI195649 |  |
| EPI195651 |  |
| EPI195653 |  |
| EPI195655 |  |
| EPI195657 |  |
| EPI195659 |  |
| EPI195661 |  |
| EPI195663 |  |
| EPI195665 |  |
| EPI195667 |  |
| EPI195669 |  |
| EPI195671 |  |
| EPI195673 |  |
| EPI195675 |  |
| EPI195677 |  |
| EPI195679 |  |
| EPI195681 |  |
| EPI195683 |  |
| EPI195685 |  |
| EPI195687 |  |
| EPI195689 |  |
| EPI195691 |  |
| EPI195693 |  |
| EPI195695 |  |
| EPI195697 |  |
| EPI195768 |  |
| EPI195776 |  |
| EPI195784 |  |
| EPI195792 |  |
| EPI195800 |  |
| EPI195808 |  |
| EPI195816 |  |
| EPI195832 |  |
| EPI195851 |  |
| EPI195854 |  |
| EPI195857 |  |
| EPI195860 |  |
| EPI195863 |  |
| EPI195870 |  |
| EPI195885 |  |
| EPI195890 |  |
| EPI195898 |  |
| EPI195905 |  |
| EPI195908 |  |
| EPI195911 |  |
| EPI195914 |  |
| EPI195917 |  |
| EPI195920 |  |
| EPI195923 |  |
| EPI195925 |  |
| EPI195930 |  |
| EPI195935 |  |
| EPI195938 |  |
| EPI195941 |  |
| EPI195944 |  |
| EPI195947 |  |
| EPI195950 |  |
| EPI195953 |  |
| EPI195956 |  |
| EPI195959 |  |
| EPI195962 |  |
| EPI195965 |  |
| EPI195968 |  |
| EPI195971 |  |
| EPI195974 |  |
| EPI195977 |  |
| EPI195980 |  |
| EPI195983 |  |
| EPI195986 |  |
| EPI195989 |  |
| EPI195992 |  |
| EPI195995 |  |
| EPI195998 |  |
| EPI196001 |  |
| EPI196004 |  |
| EPI196007 |  |
| EPI196010 |  |
| EPI196015 |  |
| EPI196020 |  |
| EPI196023 |  |
| EPI196028 |  |
| EPI196031 |  |
| EPI196034 |  |
| EPI196037 |  |
| EPI196040 |  |
| EPI196043 |  |
| EPI196046 |  |
| EPI196049 |  |
| EPI196052 |  |
| EPI196055 |  |
| EPI196058 |  |
| EPI196061 |  |
| EPI196064 |  |
| EPI196067 |  |
| EPI196070 |  |
| EPI196073 |  |
| EPI196076 |  |
| EPI196081 |  |
| EPI196084 |  |
| EPI196087 |  |
| EPI196433 |  |
| EPI196434 |  |
| EPI196435 |  |
| EPI196672 |  |
| EPI196701 |  |
| EPI196703 |  |
| EPI196705 |  |
| EPI196709 |  |
| EPI196711 |  |
| EPI196713 |  |
| EPI196716 |  |
| EPI196718 |  |
| EPI196722 |  |
| EPI196943 |  |
| EPI197000 |  |
| EPI197618 |  |
| EPI197630 |  |
| EPI197638 |  |
| EPI197811 |  |
| EPI197814 |  |
| EPI197817 |  |
| EPI197820 |  |
| EPI197823 |  |
| EPI197826 |  |
| EPI197829 |  |
| EPI197832 |  |
| EPI197835 |  |
| EPI197838 |  |
| EPI197841 |  |
| EPI197844 |  |
| EPI197847 |  |
| EPI197850 |  |
| EPI197853 |  |
| EPI197856 |  |
| EPI197859 |  |
| EPI197862 |  |
| EPI197865 |  |
| EPI197868 |  |
| EPI197871 |  |
| EPI197948 |  |
| EPI197951 |  |
| EPI197954 |  |
| EPI197957 |  |
| EPI197960 |  |
| EPI197963 |  |
| EPI197966 |  |
| EPI197969 |  |
| EPI197972 |  |
| EPI197974 |  |
| EPI197977 |  |
| EPI197980 |  |
| EPI197983 |  |
| EPI197986 |  |
| EPI198011 |  |
| EPI198019 |  |
| EPI198035 |  |
| EPI198043 |  |
| EPI198051 |  |
| EPI198059 |  |
| EPI198067 |  |
| EPI198075 |  |
| EPI198091 |  |
| EPI198099 |  |
| EPI198107 |  |
| EPI198115 |  |
| EPI198123 |  |
| EPI198131 |  |
| EPI198139 |  |
| EPI198147 |  |
| EPI198155 |  |
| EPI198163 |  |
| EPI198171 |  |
| EPI198179 |  |
| EPI198187 |  |
| EPI198195 |  |
| EPI198203 |  |
| EPI198211 |  |
| EPI198219 |  |
| EPI198227 |  |
| EPI198235 |  |
| EPI198243 |  |
| EPI198251 |  |
| EPI198259 |  |
| EPI198267 |  |
| EPI198283 |  |
| EPI198291 |  |
| EPI198299 |  |
| EPI198315 |  |
| EPI198323 |  |
| EPI198331 |  |
| EPI198339 |  |
| EPI198347 |  |
| EPI198362 |  |
| EPI198370 |  |
| EPI198378 |  |
| EPI198386 |  |
| EPI198394 |  |
| EPI198402 |  |
| EPI198410 |  |
| EPI198418 |  |
| EPI198426 |  |
| EPI198434 |  |
| EPI198442 |  |
| EPI198450 |  |
| EPI198458 |  |
| EPI198466 |  |
| EPI198474 |  |
| EPI198482 |  |
| EPI198490 |  |
| EPI198498 |  |
| EPI198506 |  |
| EPI198514 |  |
| EPI198522 |  |
| EPI198530 |  |
| EPI198538 |  |
| EPI198546 |  |
| EPI198554 |  |
| EPI198570 |  |
| EPI198578 |  |
| EPI198586 |  |
| EPI198594 |  |
| EPI198602 |  |
| EPI198610 |  |
| EPI198618 |  |
| EPI198626 |  |
| EPI198634 |  |
| EPI198642 |  |
| EPI198839 |  |
| EPI198848 |  |
| EPI198856 |  |
| EPI198871 |  |
| EPI198879 |  |
| EPI198887 |  |
| EPI198895 |  |
| EPI198903 |  |
| EPI198911 |  |
| EPI198919 |  |
| EPI198927 |  |
| EPI198935 |  |
| EPI198943 |  |
| EPI198951 |  |
| EPI198959 |  |
| EPI198967 |  |
| EPI198975 |  |
| EPI198983 |  |
| EPI198991 |  |
| EPI198999 |  |
| EPI199007 |  |
| EPI199015 |  |
| EPI199023 |  |
| EPI199031 |  |
| EPI199039 |  |
| EPI199047 |  |
| EPI199055 |  |
| EPI199063 |  |
| EPI199071 |  |
| EPI199079 |  |
| EPI199087 |  |
| EPI199095 |  |
| EPI199103 |  |
| EPI199111 |  |
| EPI199114 |  |
| EPI199126 |  |
| EPI199134 |  |
| EPI199142 |  |
| EPI199150 |  |
| EPI199158 |  |
| EPI199166 |  |
| EPI199174 |  |
| EPI199182 |  |
| EPI199190 |  |
| EPI199198 |  |
| EPI199206 |  |
| EPI199214 |  |
| EPI199222 |  |
| EPI199230 |  |
| EPI199238 |  |
| EPI199320 |  |
| EPI199323 |  |
| EPI199326 |  |
| EPI199329 |  |
| EPI199332 |  |
| EPI199335 |  |
| EPI199338 |  |
| EPI199341 |  |
| EPI199344 |  |
| EPI199347 |  |
| EPI199360 |  |
| EPI199368 |  |
| EPI199403 |  |
| EPI199425 |  |
| EPI199433 |  |
| EPI199441 |  |
| EPI199449 |  |
| EPI199454 |  |
| EPI199462 |  |
| EPI199470 |  |
| EPI199486 |  |
| EPI199494 |  |
| EPI199502 |  |
| EPI199510 |  |
| EPI199518 |  |
| EPI199526 |  |
| EPI199960 |  |
| EPI199968 |  |
| EPI199976 |  |
| EPI199984 |  |
| EPI199992 |  |
| EPI200000 |  |
| EPI200008 |  |
| EPI200016 |  |
| EPI200024 |  |
| EPI200032 |  |
| EPI200040 |  |
| EPI200048 |  |
| EPI200056 |  |
| EPI200064 |  |
| EPI200072 |  |
| EPI200080 |  |
| EPI200088 |  |
| EPI200100 |  |
| EPI200108 |  |
| EPI200116 |  |
| EPI200124 |  |
| EPI200308 |  |
| EPI200315 |  |
| EPI200327 |  |
| EPI200336 |  |
| EPI200342 |  |
| EPI200351 |  |
| EPI200360 |  |
| EPI200363 |  |
| EPI201650 |  |
| EPI201711 |  |
| EPI201782 |  |
| EPI201784 |  |
| EPI201788 |  |
| EPI201794 |  |
| EPI201923 |  |
| EPI201931 |  |
| EPI201950 |  |
| EPI201994 |  |
| EPI215958 |  |
| EPI215960 |  |
| EPI215962 |  |
| EPI215964 |  |
| EPI215966 |  |
| EPI215968 |  |
| EPI215970 |  |
| EPI215972 |  |
| EPI215974 |  |
| EPI215976 |  |
| EPI215978 |  |
| EPI216576 |  |
| EPI216584 |  |
| EPI216592 |  |
| EPI216595 |  |
| EPI216603 |  |
| EPI216611 |  |
| EPI216619 |  |
| EPI216627 |  |
| EPI216635 |  |
| EPI216643 |  |
| EPI216651 |  |
| EPI216659 |  |
| EPI216667 |  |
| EPI216675 |  |
| EPI216683 |  |
| EPI216691 |  |
| EPI216693 |  |
| EPI216696 |  |
| EPI216699 |  |
| EPI216707 |  |
| EPI216714 |  |
| EPI216717 |  |
| EPI216720 |  |
| EPI216723 |  |
| EPI216726 |  |
| EPI216729 |  |
| EPI216732 |  |
| EPI216735 |  |
| EPI216744 |  |
| EPI216747 |  |
| EPI216750 |  |
| EPI216753 |  |
| EPI216755 |  |
| EPI216758 |  |
| EPI216766 |  |
| EPI216774 |  |
| EPI216777 |  |
| EPI216780 |  |
| EPI216783 |  |
| EPI216786 |  |
| EPI216789 |  |
| EPI216797 |  |
| EPI216805 |  |
| EPI216813 |  |
| EPI216821 |  |
| EPI216836 |  |
| EPI216841 |  |
| EPI216844 |  |
| EPI216847 |  |
| EPI216850 |  |
| EPI216853 |  |
| EPI216856 |  |
| EPI216859 |  |
| EPI216862 |  |
| EPI216865 |  |
| EPI216868 |  |
| EPI216871 |  |
| EPI216876 |  |
| EPI216879 |  |
| EPI216885 |  |
| EPI216888 |  |
| EPI216896 |  |
| EPI216904 |  |
| EPI216912 |  |
| EPI216920 |  |
| EPI216928 |  |
| EPI216931 |  |
| EPI216934 |  |
| EPI216937 |  |
| EPI216940 |  |
| EPI216948 |  |
| EPI216956 |  |
| EPI216959 |  |
| EPI216963 |  |
| EPI216966 |  |
| EPI216973 |  |
| EPI216976 |  |
| EPI216978 |  |
| EPI216981 |  |
| EPI216984 |  |
| EPI216991 |  |
| EPI216994 |  |
| EPI216997 |  |
| EPI217000 |  |
| EPI217003 |  |
| EPI217007 |  |
| EPI217010 |  |
| EPI217013 |  |
| EPI217016 |  |
| EPI217019 |  |
| EPI217024 |  |
| EPI217027 |  |
| EPI217034 |  |
| EPI217041 |  |
| EPI217047 |  |
| EPI217050 |  |
| EPI217057 |  |
| EPI217062 |  |
| EPI217065 |  |
| EPI217068 |  |
| EPI217071 |  |
| EPI217074 |  |
| EPI217076 |  |
| EPI217078 |  |
| EPI217081 |  |
| EPI217084 |  |
| EPI217087 |  |
| EPI217090 |  |
| EPI217093 |  |
| EPI217096 |  |
| EPI217110 |  |
| EPI217113 |  |
| EPI217116 |  |
| EPI217119 |  |
| EPI217122 |  |
| EPI217125 |  |
| EPI217128 |  |
| EPI217131 |  |
| EPI217134 |  |
| EPI217137 |  |
| EPI217140 |  |
| EPI217143 |  |
| EPI217146 |  |
| EPI217149 |  |
| EPI217152 |  |
| EPI217155 |  |
| EPI217158 |  |
| EPI217161 |  |
| EPI217166 |  |
| EPI217169 |  |
| EPI217172 |  |
| EPI217177 |  |
| EPI217180 |  |
| EPI217185 |  |
| EPI217188 |  |
| EPI217191 |  |
| EPI217194 |  |
| EPI217202 |  |
| EPI217205 |  |
| EPI217208 |  |
| EPI217211 |  |
| EPI217214 |  |
| EPI217217 |  |
| EPI217220 |  |
| EPI217223 |  |
| EPI217230 |  |
| EPI217233 |  |
| EPI217239 |  |
| EPI217242 |  |
| EPI217245 |  |
| EPI217248 |  |
| EPI217251 |  |
| EPI217254 |  |
| EPI217257 |  |
| EPI217259 |  |
| EPI217262 |  |
| EPI217265 |  |
| EPI217268 |  |
| EPI217271 |  |
| EPI217337 |  |
| EPI217342 |  |
| EPI217397 |  |
| EPI217403 |  |
| EPI217406 |  |
| EPI217409 |  |
| EPI217415 |  |
| EPI217418 |  |
| EPI217424 |  |
| EPI217430 |  |
| EPI217433 |  |
| EPI217439 |  |
| EPI217442 |  |
| EPI218343 |  |
| EPI218366 |  |
| EPI218372 |  |
| EPI218407 |  |
| EPI218537 |  |
| EPI218548 |  |
| EPI219414 |  |
| EPI219440 |  |
| EPI219442 |  |
| EPI219444 |  |
| EPI219446 |  |
| EPI219447 |  |
| EPI219448 |  |
| EPI219449 |  |
| EPI219450 |  |
| EPI220610 |  |
| EPI220960 |  |
| EPI220961 |  |
| EPI220971 |  |
| EPI220974 |  |
| EPI220977 |  |
| EPI220995 |  |
| EPI221013 |  |
| EPI221029 |  |
| EPI221035 |  |
| EPI221043 |  |
| EPI221068 |  |
| EPI221080 |  |
| EPI221090 |  |
| EPI221094 |  |
| EPI221121 |  |
| EPI221125 |  |
| EPI221163 |  |
| EPI221171 |  |
| EPI221186 |  |
| EPI221214 |  |
| EPI221224 |  |
| EPI221230 |  |
| EPI221277 |  |
| EPI221281 |  |
| EPI221287 |  |
| EPI221291 |  |
| EPI221293 |  |
| EPI221346 |  |
| EPI221347 |  |
| EPI221348 |  |
| EPI221349 |  |
| EPI221352 |  |
| EPI221372 |  |
| EPI221373 |  |
| EPI221374 |  |
| EPI221375 |  |
| EPI221376 |  |
| EPI222819 |  |
| EPI222820 |  |
| EPI222821 |  |
| EPI222822 |  |
| EPI222823 |  |
| EPI222824 |  |
| EPI222825 |  |
| EPI222826 |  |
| EPI222827 |  |
| EPI222829 |  |
| EPI222831 |  |
| EPI222832 |  |
| EPI222981 |  |
| EPI222989 |  |
| EPI223142 |  |
| EPI223143 |  |
| EPI223145 |  |
| EPI223149 |  |
| EPI223152 |  |
| EPI223157 |  |
| EPI223187 |  |
| EPI223195 |  |
| EPI223203 |  |
| EPI223211 |  |
| EPI223219 |  |
| EPI223227 |  |
| EPI223235 |  |
| EPI223243 |  |
| EPI223251 |  |
| EPI223259 |  |
| EPI223267 |  |
| EPI223275 |  |
| EPI223283 |  |
| EPI223291 |  |
| EPI223299 |  |
| EPI223307 |  |
| EPI223315 |  |
| EPI223323 |  |
| EPI223331 |  |
| EPI223339 |  |
| EPI223347 |  |
| EPI223355 |  |
| EPI223363 |  |
| EPI223371 |  |
| EPI223379 |  |
| EPI223387 |  |
| EPI223395 |  |
| EPI223403 |  |
| EPI223411 |  |
| EPI223419 |  |
| EPI223442 |  |
| EPI223450 |  |
| EPI223458 |  |
| EPI223466 |  |
| EPI223474 |  |
| EPI223482 |  |
| EPI223490 |  |
| EPI223498 |  |
| EPI223506 |  |
| EPI223514 |  |
| EPI223522 |  |
| EPI223530 |  |
| EPI223538 |  |
| EPI223546 |  |
| EPI223554 |  |
| EPI223562 |  |
| EPI223570 |  |
| EPI223578 |  |
| EPI223586 |  |
| EPI223594 |  |
| EPI223602 |  |
| EPI223610 |  |
| EPI223618 |  |
| EPI223626 |  |
| EPI223634 |  |
| EPI223642 |  |
| EPI223650 |  |
| EPI223658 |  |
| EPI223666 |  |
| EPI223674 |  |
| EPI223682 |  |
| EPI223690 |  |
| EPI223698 |  |
| EPI223706 |  |
| EPI223714 |  |
| EPI223722 |  |
| EPI223730 |  |
| EPI223738 |  |
| EPI223746 |  |
| EPI223754 |  |
| EPI223762 |  |
| EPI223770 |  |
| EPI223778 |  |
| EPI223786 |  |
| EPI223794 |  |
| EPI223802 |  |
| EPI223810 |  |
| EPI223818 |  |
| EPI223826 |  |
| EPI223834 |  |
| EPI223842 |  |
| EPI223850 |  |
| EPI223858 |  |
| EPI223866 |  |
| EPI223874 |  |
| EPI223882 |  |
| EPI223890 |  |
| EPI223898 |  |
| EPI223906 |  |
| EPI223914 |  |
| EPI223922 |  |
| EPI223930 |  |
| EPI223938 |  |
| EPI223946 |  |
| EPI223954 |  |
| EPI223962 |  |
| EPI223970 |  |
| EPI223978 |  |
| EPI223986 |  |
| EPI223994 |  |
| EPI224002 |  |
| EPI224010 |  |
| EPI224018 |  |
| EPI224026 |  |
| EPI224034 |  |
| EPI224050 |  |
| EPI224058 |  |
| EPI224066 |  |
| EPI224074 |  |
| EPI224082 |  |
| EPI224090 |  |
| EPI224098 |  |
| EPI224106 |  |
| EPI224114 |  |
| EPI224122 |  |
| EPI224130 |  |
| EPI224138 |  |
| EPI224146 |  |
| EPI224154 |  |
| EPI224162 |  |
| EPI224170 |  |
| EPI224178 |  |
| EPI224186 |  |
| EPI224194 |  |
| EPI224202 |  |
| EPI224210 |  |
| EPI224218 |  |
| EPI224226 |  |
| EPI224234 |  |
| EPI224242 |  |
| EPI224250 |  |
| EPI224258 |  |
| EPI224266 |  |
| EPI224274 |  |
| EPI224282 |  |
| EPI224290 |  |
| EPI224298 |  |
| EPI224306 |  |
| EPI224314 |  |
| EPI224322 |  |
| EPI224330 |  |
| EPI224338 |  |
| EPI224346 |  |
| EPI224354 |  |
| EPI224362 |  |
| EPI224370 |  |
| EPI224376 |  |
| EPI224378 |  |
| EPI224380 |  |
| EPI224383 |  |
| EPI224385 |  |
| EPI224387 |  |
| EPI224391 |  |
| EPI224393 |  |
| EPI224395 |  |
| EPI224397 |  |
| EPI224399 |  |
| EPI224406 |  |
| EPI224412 |  |
| EPI224418 |  |
| EPI224424 |  |
| EPI224428 |  |
| EPI224433 |  |
| EPI224439 |  |
| EPI224445 |  |
| EPI225847 |  |
| EPI225855 |  |
| EPI225863 |  |
| EPI225871 |  |
| EPI225879 |  |
| EPI225887 |  |
| EPI225895 |  |
| EPI225903 |  |
| EPI225911 |  |
| EPI225919 |  |
| EPI225927 |  |
| EPI225935 |  |
| EPI225943 |  |
| EPI225951 |  |
| EPI225959 |  |
| EPI225967 |  |
| EPI225975 |  |
| EPI225983 |  |
| EPI225991 |  |
| EPI225999 |  |
| EPI226007 |  |
| EPI226015 |  |
| EPI226023 |  |
| EPI226031 |  |
| EPI226039 |  |
| EPI226047 |  |
| EPI226055 |  |
| EPI226063 |  |
| EPI226071 |  |
| EPI226079 |  |
| EPI226087 |  |
| EPI226095 |  |
| EPI226103 |  |
| EPI226111 |  |
| EPI226119 |  |
| EPI226127 |  |
| EPI226135 |  |
| EPI226143 |  |
| EPI226151 |  |
| EPI226159 |  |
| EPI226167 |  |
| EPI226175 |  |
| EPI226183 |  |
| EPI226191 |  |
| EPI226199 |  |
| EPI226207 |  |
| EPI226215 |  |
| EPI226223 |  |
| EPI226231 |  |
| EPI226239 |  |
| EPI226247 |  |
| EPI226255 |  |
| EPI226263 |  |
| EPI226271 |  |
| EPI226279 |  |
| EPI226287 |  |
| EPI226295 |  |
| EPI226303 |  |
| EPI226311 |  |
| EPI226319 |  |
| EPI226327 |  |
| EPI226335 |  |
| EPI226343 |  |
| EPI226351 |  |
| EPI226359 |  |
| EPI226367 |  |
| EPI226375 |  |
| EPI226383 |  |
| EPI226391 |  |
| EPI226399 |  |
| EPI226407 |  |
| EPI226415 |  |
| EPI226423 |  |
| EPI226431 |  |
| EPI226439 |  |
| EPI226447 |  |
| EPI226455 |  |
| EPI226463 |  |
| EPI226471 |  |
| EPI226479 |  |
| EPI226487 |  |
| EPI226495 |  |
| EPI226503 |  |
| EPI226511 |  |
| EPI226519 |  |
| EPI226527 |  |
| EPI226543 |  |
| EPI226559 |  |
| EPI226567 |  |
| EPI226575 |  |
| EPI226583 |  |
| EPI226591 |  |
| EPI226599 |  |
| EPI227617 |  |
| EPI227621 |  |
| EPI227624 |  |
| EPI227627 |  |
| EPI227631 |  |
| EPI227639 |  |
| EPI227649 |  |
| EPI227652 |  |
| EPI227654 |  |
| EPI227656 |  |
| EPI227658 |  |
| EPI227659 |  |
| EPI227662 |  |
| EPI227665 |  |
| EPI227675 |  |
| EPI227680 |  |
| EPI227683 |  |
| EPI227689 |  |
| EPI227692 |  |
| EPI227694 |  |
| EPI227704 |  |
| EPI227707 |  |
| EPI227715 |  |
| EPI227722 |  |
| EPI227724 |  |
| EPI227727 |  |
| EPI227730 |  |
| EPI227733 |  |
| EPI227740 |  |
| EPI227744 |  |
| EPI227748 |  |
| EPI227750 |  |
| EPI227758 |  |
| EPI227761 |  |
| EPI227769 |  |
| EPI227776 |  |
| EPI227784 |  |
| EPI227791 |  |
| EPI227794 |  |
| EPI227802 |  |
| EPI227810 |  |
| EPI227813 |  |
| EPI227819 |  |
| EPI227821 |  |
| EPI227823 |  |
| EPI227826 |  |
| EPI227832 |  |
| EPI227835 |  |
| EPI227840 |  |
| EPI227844 |  |
| EPI227847 |  |
| EPI227855 |  |
| EPI227863 |  |
| EPI227869 |  |
| EPI227874 |  |
| EPI227880 |  |
| EPI227882 |  |
| EPI227885 |  |
| EPI227893 |  |
| EPI227899 |  |
| EPI227901 |  |
| EPI227909 |  |
| EPI227911 |  |
| EPI227916 |  |
| EPI227918 |  |
| EPI227919 |  |
| EPI227926 |  |
| EPI227929 |  |
| EPI227934 |  |
| EPI228206 |  |
| EPI228211 |  |
| EPI228307 |  |
| EPI228309 |  |
| EPI229722 |  |
| EPI229726 |  |
| EPI229729 |  |
| EPI229759 |  |
| EPI229767 |  |
| EPI229775 |  |
| EPI229783 |  |
| EPI229791 |  |
| EPI230044 |  |
| EPI230046 |  |
| EPI230048 |  |
| EPI230050 |  |
| EPI230054 |  |
| EPI230056 |  |
| EPI230060 |  |
| EPI230062 |  |
| EPI230065 |  |
| EPI230068 |  |
| EPI230076 |  |
| EPI230078 |  |
| EPI230082 |  |
| EPI230084 |  |
| EPI230086 |  |
| EPI230088 |  |
| EPI230090 |  |
| EPI230092 |  |
| EPI230094 |  |
| EPI230096 |  |
| EPI230098 |  |
| EPI230100 |  |
| EPI230102 |  |
| EPI230104 |  |
| EPI230106 |  |
| EPI230108 |  |
| EPI230110 |  |
| EPI230112 |  |
| EPI230114 |  |
| EPI230116 |  |
| EPI230118 |  |
| EPI230120 |  |
| EPI230209 |  |
| EPI230214 |  |
| EPI230274 |  |
| EPI230279 |  |
| EPI230283 |  |
| EPI230289 |  |
| EPI230295 |  |
| EPI230303 |  |
| EPI230311 |  |
| EPI230321 |  |
| EPI230343 |  |
| EPI230353 |  |
| EPI230364 |  |
| EPI230370 |  |
| EPI230376 |  |
| EPI230379 |  |
| EPI230388 |  |
| EPI230402 |  |
| EPI230410 |  |
| EPI230418 |  |
| EPI230426 |  |
| EPI230439 |  |
| EPI230443 |  |
| EPI230446 |  |
| EPI230450 |  |
| EPI230454 |  |
| EPI230458 |  |
| EPI230462 |  |
| EPI230466 |  |
| EPI230483 |  |
| EPI230491 |  |
| EPI230499 |  |
| EPI230507 |  |
