# Supplementary material for: Selection for resistance to oseltamivir in seasonal and pandemic H1N1 influenza and widespread co-circulation of the lineages
Source: Int J Health Geogr. 2010 Feb 24;9:13. doi: 10.1186/1476-072X-9-13 (PMC2882220; doi:10.1186/1476-072X-9-13)
Supplement: Additional file 4 — Accession numbers of the neuraminidase nucleotide sequences used in the phylogenetic and geographic study of seasonal H1N1 influenza A. GISAID sequences are available at http://www.gisaid.org. GenBank sequences are available at http://ncbi.nlm.nih.gov. [file 1476-072X-9-13-S4.DOC]

| GISAID | GenBank |
| --- | --- |
| EPI182496 | AB509160 |
| EPI120070 | AB509161 |
| EPI123094 | AB509162 |
| EPI123110 | AB509163 |
| EPI123118 | AB509164 |
| EPI125969 | CY050478 |
| EPI126005 | CY050550 |
| EPI133912 | CY050614 |
| EPI133930 | CY050662 |
| EPI133932 | CY050750 |
| EPI133934 | CY050758 |
| EPI133936 | CY050766 |
| EPI133938 | CY050774 |
| EPI133942 | CY053651 |
| EPI133950 | CY053655 |
| EPI133958 | CY053657 |
| EPI133962 | CY053659 |
| EPI133968 | CY053665 |
| EPI133976 | CY053671 |
| EPI133988 | CY053677 |
| EPI133992 | GQ902803 |
| EPI133994 | GQ902835 |
| EPI134058 | GQ994963 |
| EPI134124 | GQ994964 |
| EPI134126 | GQ994965 |
| EPI134128 | GQ994966 |
| EPI134130 | GQ994967 |
| EPI134132 | GQ994968 |
| EPI134134 | GQ994969 |
| EPI134136 | GQ994970 |
| EPI134138 | GQ994971 |
| EPI134140 | GU112228 |
| EPI134144 | GU112229 |
| EPI134146 | GU112230 |
| EPI134150 | GU183801 |
| EPI134152 | GU183809 |
| EPI134156 | GU183817 |
| EPI134419 | taxid461787 |
| EPI134441 | taxid521433 |
| EPI134493 | taxid530146 |
| EPI134501 | taxid530163 |
| EPI134531 | taxid569644 |
| EPI134533 | taxid569645 |
| EPI134535 | taxid569675 |
| EPI134537 | taxid569677 |
| EPI134539 | taxid569678 |
| EPI134541 | taxid569679 |
| EPI134545 | taxid569681 |
| EPI134547 | taxid569682 |
| EPI134549 | taxid569684 |
| EPI134551 | taxid569688 |
| EPI137847 | taxid569692 |
| EPI137851 | taxid569693 |
| EPI137855 | taxid569695 |
| EPI137859 | taxid569696 |
| EPI138744 | taxid569697 |
| EPI138750 | taxid569704 |
| EPI138753 | taxid569709 |
| EPI138754 | taxid569711 |
| EPI138756 | taxid591294 |
| EPI138759 | taxid591295 |
| EPI138763 | taxid638927 |
| EPI138768 | taxid638928 |
| EPI138772 |  |
| EPI138774 |  |
| EPI138775 |  |
| EPI138777 |  |
| EPI138778 |  |
| EPI138779 |  |
| EPI138781 |  |
| EPI138782 |  |
| EPI138785 |  |
| EPI138787 |  |
| EPI138793 |  |
| EPI138794 |  |
| EPI138853 |  |
| EPI138909 |  |
| EPI141223 |  |
| EPI141224 |  |
| EPI141225 |  |
| EPI141226 |  |
| EPI141227 |  |
| EPI141302 |  |
| EPI142382 |  |
| EPI152648 |  |
| EPI152650 |  |
| EPI152651 |  |
| EPI152654 |  |
| EPI152658 |  |
| EPI152659 |  |
| EPI152662 |  |
| EPI152683 |  |
| EPI152695 |  |
| EPI152698 |  |
| EPI152699 |  |
| EPI152702 |  |
| EPI152712 |  |
| EPI152720 |  |
| EPI152722 |  |
| EPI152723 |  |
| EPI152738 |  |
| EPI153103 |  |
| EPI153104 |  |
| EPI153105 |  |
| EPI153106 |  |
| EPI153107 |  |
| EPI154304 |  |
| EPI154305 |  |
| EPI154306 |  |
| EPI154307 |  |
| EPI154308 |  |
| EPI154309 |  |
| EPI154310 |  |
| EPI154311 |  |
| EPI154312 |  |
| EPI154314 |  |
| EPI154315 |  |
| EPI154316 |  |
| EPI154317 |  |
| EPI154318 |  |
| EPI154319 |  |
| EPI154320 |  |
| EPI154321 |  |
| EPI154322 |  |
| EPI154323 |  |
| EPI154324 |  |
| EPI154326 |  |
| EPI155268 |  |
| EPI155275 |  |
| EPI155277 |  |
| EPI155281 |  |
| EPI155536 |  |
| EPI155541 |  |
| EPI155561 |  |
| EPI155706 |  |
| EPI155895 |  |
| EPI155900 |  |
| EPI156108 |  |
| EPI156110 |  |
| EPI156188 |  |
| EPI156190 |  |
| EPI156194 |  |
| EPI156196 |  |
| EPI156197 |  |
| EPI156199 |  |
| EPI156200 |  |
| EPI157481 |  |
| EPI157874 |  |
| EPI157880 |  |
| EPI157884 |  |
| EPI157929 |  |
| EPI157931 |  |
| EPI157968 |  |
| EPI157970 |  |
| EPI157995 |  |
| EPI157998 |  |
| EPI158047 |  |
| EPI158084 |  |
| EPI158087 |  |
| EPI158202 |  |
| EPI158234 |  |
| EPI158238 |  |
| EPI158241 |  |
| EPI158263 |  |
| EPI159863 |  |
| EPI159864 |  |
| EPI159866 |  |
| EPI159867 |  |
| EPI159874 |  |
| EPI159879 |  |
| EPI159881 |  |
| EPI160250 |  |
| EPI160252 |  |
| EPI160253 |  |
| EPI160254 |  |
| EPI160255 |  |
| EPI160256 |  |
| EPI160259 |  |
| EPI160261 |  |
| EPI160262 |  |
| EPI160263 |  |
| EPI160264 |  |
| EPI160265 |  |
| EPI160266 |  |
| EPI160267 |  |
| EPI160269 |  |
| EPI160271 |  |
| EPI160309 |  |
| EPI160310 |  |
| EPI161657 |  |
| EPI161658 |  |
| EPI161659 |  |
| EPI161660 |  |
| EPI161661 |  |
| EPI161662 |  |
| EPI161663 |  |
| EPI161664 |  |
| EPI161837 |  |
| EPI161843 |  |
| EPI161875 |  |
| EPI161890 |  |
| EPI161892 |  |
| EPI161913 |  |
| EPI161919 |  |
| EPI161993 |  |
| EPI161995 |  |
| EPI162013 |  |
| EPI162073 |  |
| EPI162083 |  |
| EPI162087 |  |
| EPI162103 |  |
| EPI162142 |  |
| EPI162167 |  |
| EPI162191 |  |
| EPI162193 |  |
| EPI162195 |  |
| EPI162197 |  |
| EPI162199 |  |
| EPI162207 |  |
| EPI162209 |  |
| EPI162215 |  |
| EPI162217 |  |
| EPI162221 |  |
| EPI162223 |  |
| EPI162225 |  |
| EPI162227 |  |
| EPI162229 |  |
| EPI162231 |  |
| EPI162233 |  |
| EPI162235 |  |
| EPI162238 |  |
| EPI162263 |  |
| EPI162278 |  |
| EPI162290 |  |
| EPI162293 |  |
| EPI162332 |  |
| EPI162622 |  |
| EPI162623 |  |
| EPI162625 |  |
| EPI162626 |  |
| EPI162632 |  |
| EPI162634 |  |
| EPI162643 |  |
| EPI162647 |  |
| EPI162650 |  |
| EPI162653 |  |
| EPI162662 |  |
| EPI162665 |  |
| EPI162668 |  |
| EPI162669 |  |
| EPI162674 |  |
| EPI162675 |  |
| EPI162678 |  |
| EPI162679 |  |
| EPI162694 |  |
| EPI162695 |  |
| EPI162699 |  |
| EPI162701 |  |
| EPI162703 |  |
| EPI162707 |  |
| EPI162712 |  |
| EPI162727 |  |
| EPI162729 |  |
| EPI162732 |  |
| EPI162733 |  |
| EPI162734 |  |
| EPI162735 |  |
| EPI162737 |  |
| EPI162743 |  |
| EPI162745 |  |
| EPI162748 |  |
| EPI162749 |  |
| EPI162752 |  |
| EPI162760 |  |
| EPI162770 |  |
| EPI162771 |  |
| EPI162772 |  |
| EPI162776 |  |
| EPI162783 |  |
| EPI162785 |  |
| EPI162786 |  |
| EPI162788 |  |
| EPI162805 |  |
| EPI162806 |  |
| EPI162808 |  |
| EPI162810 |  |
| EPI162812 |  |
| EPI162815 |  |
| EPI162817 |  |
| EPI162960 |  |
| EPI162984 |  |
| EPI162997 |  |
| EPI163001 |  |
| EPI163003 |  |
| EPI163007 |  |
| EPI163009 |  |
| EPI163011 |  |
| EPI163013 |  |
| EPI163015 |  |
| EPI163019 |  |
| EPI163021 |  |
| EPI163023 |  |
| EPI163025 |  |
| EPI163029 |  |
| EPI163031 |  |
| EPI163033 |  |
| EPI163035 |  |
| EPI163037 |  |
| EPI163039 |  |
| EPI163041 |  |
| EPI163043 |  |
| EPI163045 |  |
| EPI163047 |  |
| EPI163049 |  |
| EPI163051 |  |
| EPI163053 |  |
| EPI163055 |  |
| EPI163057 |  |
| EPI163059 |  |
| EPI163061 |  |
| EPI163064 |  |
| EPI163066 |  |
| EPI163070 |  |
| EPI163071 |  |
| EPI163072 |  |
| EPI163074 |  |
| EPI163076 |  |
| EPI163078 |  |
| EPI163080 |  |
| EPI163082 |  |
| EPI163083 |  |
| EPI163087 |  |
| EPI163199 |  |
| EPI163201 |  |
| EPI163203 |  |
| EPI163205 |  |
| EPI163207 |  |
| EPI163209 |  |
| EPI163210 |  |
| EPI163212 |  |
| EPI163216 |  |
| EPI163218 |  |
| EPI163220 |  |
| EPI163222 |  |
| EPI163224 |  |
| EPI163226 |  |
| EPI163244 |  |
| EPI163245 |  |
| EPI165080 |  |
| EPI165248 |  |
| EPI165250 |  |
| EPI165251 |  |
| EPI165252 |  |
| EPI165253 |  |
| EPI165256 |  |
| EPI165262 |  |
| EPI165263 |  |
| EPI165264 |  |
| EPI165265 |  |
| EPI165268 |  |
| EPI165269 |  |
| EPI165270 |  |
| EPI165271 |  |
| EPI165272 |  |
| EPI165273 |  |
| EPI165274 |  |
| EPI165275 |  |
| EPI165276 |  |
| EPI165277 |  |
| EPI165278 |  |
| EPI165279 |  |
| EPI165280 |  |
| EPI165281 |  |
| EPI165282 |  |
| EPI165283 |  |
| EPI165284 |  |
| EPI165285 |  |
| EPI165286 |  |
| EPI165287 |  |
| EPI165288 |  |
| EPI165289 |  |
| EPI165290 |  |
| EPI165292 |  |
| EPI165293 |  |
| EPI165295 |  |
| EPI165297 |  |
| EPI165298 |  |
| EPI165302 |  |
| EPI165304 |  |
| EPI165305 |  |
| EPI165306 |  |
| EPI165307 |  |
| EPI165308 |  |
| EPI165309 |  |
| EPI165310 |  |
| EPI165311 |  |
| EPI165727 |  |
| EPI165728 |  |
| EPI165729 |  |
| EPI166027 |  |
| EPI166028 |  |
| EPI166029 |  |
| EPI166030 |  |
| EPI166038 |  |
| EPI166039 |  |
| EPI166075 |  |
| EPI166663 |  |
| EPI166665 |  |
| EPI166668 |  |
| EPI166672 |  |
| EPI166676 |  |
| EPI166677 |  |
| EPI166679 |  |
| EPI166681 |  |
| EPI166682 |  |
| EPI166684 |  |
| EPI166690 |  |
| EPI166693 |  |
| EPI166694 |  |
| EPI166699 |  |
| EPI166701 |  |
| EPI168073 |  |
| EPI168074 |  |
| EPI168088 |  |
| EPI168101 |  |
| EPI168113 |  |
| EPI168129 |  |
| EPI168136 |  |
| EPI168660 |  |
| EPI169204 |  |
| EPI169206 |  |
| EPI169212 |  |
| EPI169227 |  |
| EPI169229 |  |
| EPI169231 |  |
| EPI169233 |  |
| EPI169237 |  |
| EPI169241 |  |
| EPI169564 |  |
| EPI169571 |  |
| EPI169572 |  |
| EPI169573 |  |
| EPI169574 |  |
| EPI169575 |  |
| EPI169576 |  |
| EPI169577 |  |
| EPI169578 |  |
| EPI170840 |  |
| EPI170841 |  |
| EPI170842 |  |
| EPI170843 |  |
| EPI171222 |  |
| EPI171223 |  |
| EPI171432 |  |
| EPI171445 |  |
| EPI171447 |  |
| EPI171448 |  |
| EPI171449 |  |
| EPI171450 |  |
| EPI171451 |  |
| EPI171452 |  |
| EPI171453 |  |
| EPI171454 |  |
| EPI171455 |  |
| EPI171456 |  |
| EPI171457 |  |
| EPI171458 |  |
| EPI171459 |  |
| EPI171460 |  |
| EPI171461 |  |
| EPI171462 |  |
| EPI171463 |  |
| EPI171464 |  |
| EPI171465 |  |
| EPI171467 |  |
| EPI171468 |  |
| EPI171469 |  |
| EPI171470 |  |
| EPI171471 |  |
| EPI171472 |  |
| EPI171473 |  |
| EPI171474 |  |
| EPI171475 |  |
| EPI171980 |  |
| EPI171981 |  |
| EPI171982 |  |
| EPI171983 |  |
| EPI171984 |  |
| EPI171985 |  |
| EPI171986 |  |
| EPI171987 |  |
| EPI171988 |  |
| EPI171989 |  |
| EPI171990 |  |
| EPI171991 |  |
| EPI171992 |  |
| EPI171993 |  |
| EPI171994 |  |
| EPI171995 |  |
| EPI171996 |  |
| EPI172152 |  |
| EPI172153 |  |
| EPI172154 |  |
| EPI172155 |  |
| EPI172156 |  |
| EPI172157 |  |
| EPI172158 |  |
| EPI172363 |  |
| EPI172365 |  |
| EPI172366 |  |
| EPI172367 |  |
| EPI172368 |  |
| EPI172369 |  |
| EPI172372 |  |
| EPI172373 |  |
| EPI172374 |  |
| EPI172375 |  |
| EPI172376 |  |
| EPI172379 |  |
| EPI172380 |  |
| EPI172381 |  |
| EPI172385 |  |
| EPI172386 |  |
| EPI172387 |  |
| EPI172388 |  |
| EPI172389 |  |
| EPI172390 |  |
| EPI172391 |  |
| EPI172392 |  |
| EPI172393 |  |
| EPI172397 |  |
| EPI172398 |  |
| EPI172399 |  |
| EPI172400 |  |
| EPI172401 |  |
| EPI172402 |  |
| EPI172403 |  |
| EPI172404 |  |
| EPI172405 |  |
| EPI172406 |  |
| EPI172407 |  |
| EPI172574 |  |
| EPI172580 |  |
| EPI172581 |  |
| EPI172582 |  |
| EPI172583 |  |
| EPI172584 |  |
| EPI172585 |  |
| EPI172594 |  |
| EPI172595 |  |
| EPI172600 |  |
| EPI172601 |  |
| EPI172603 |  |
| EPI172604 |  |
| EPI172605 |  |
| EPI172607 |  |
| EPI172608 |  |
| EPI172609 |  |
| EPI172612 |  |
| EPI172613 |  |
| EPI172614 |  |
| EPI172615 |  |
| EPI172616 |  |
| EPI172617 |  |
| EPI172620 |  |
| EPI172621 |  |
| EPI172623 |  |
| EPI172624 |  |
| EPI172628 |  |
| EPI172631 |  |
| EPI172632 |  |
| EPI172633 |  |
| EPI172634 |  |
| EPI172646 |  |
| EPI172647 |  |
| EPI172649 |  |
| EPI172650 |  |
| EPI172652 |  |
| EPI172654 |  |
| EPI172655 |  |
| EPI172657 |  |
| EPI172659 |  |
| EPI172660 |  |
| EPI172662 |  |
| EPI172663 |  |
| EPI172664 |  |
| EPI172666 |  |
| EPI172668 |  |
| EPI172669 |  |
| EPI172671 |  |
| EPI172673 |  |
| EPI172676 |  |
| EPI172677 |  |
| EPI172679 |  |
| EPI172680 |  |
| EPI172682 |  |
| EPI172683 |  |
| EPI172684 |  |
| EPI172685 |  |
| EPI172687 |  |
| EPI172689 |  |
| EPI172691 |  |
| EPI172692 |  |
| EPI172693 |  |
| EPI172694 |  |
| EPI172695 |  |
| EPI172697 |  |
| EPI172705 |  |
| EPI172707 |  |
| EPI172709 |  |
| EPI172710 |  |
| EPI172711 |  |
| EPI172712 |  |
| EPI172723 |  |
| EPI172726 |  |
| EPI172729 |  |
| EPI172731 |  |
| EPI172732 |  |
| EPI172734 |  |
| EPI172736 |  |
| EPI172737 |  |
| EPI172738 |  |
| EPI172739 |  |
| EPI172740 |  |
| EPI172741 |  |
| EPI172742 |  |
| EPI172747 |  |
| EPI172748 |  |
| EPI172749 |  |
| EPI172751 |  |
| EPI172753 |  |
| EPI172756 |  |
| EPI172757 |  |
| EPI172759 |  |
| EPI172761 |  |
| EPI172763 |  |
| EPI172765 |  |
| EPI172766 |  |
| EPI172767 |  |
| EPI172771 |  |
| EPI172772 |  |
| EPI172804 |  |
| EPI172806 |  |
| EPI172808 |  |
| EPI172810 |  |
| EPI172812 |  |
| EPI172814 |  |
| EPI172816 |  |
| EPI172818 |  |
| EPI172820 |  |
| EPI172822 |  |
| EPI172824 |  |
| EPI172826 |  |
| EPI172828 |  |
| EPI172830 |  |
| EPI172832 |  |
| EPI172834 |  |
| EPI172836 |  |
| EPI172838 |  |
| EPI172840 |  |
| EPI172843 |  |
| EPI172845 |  |
| EPI173107 |  |
| EPI173237 |  |
| EPI173253 |  |
| EPI173256 |  |
| EPI173257 |  |
| EPI173258 |  |
| EPI173259 |  |
| EPI173260 |  |
| EPI173261 |  |
| EPI173262 |  |
| EPI173263 |  |
| EPI173264 |  |
| EPI173266 |  |
| EPI173268 |  |
| EPI173270 |  |
| EPI173283 |  |
| EPI173285 |  |
| EPI173286 |  |
| EPI173287 |  |
| EPI173288 |  |
| EPI173290 |  |
| EPI173291 |  |
| EPI173292 |  |
| EPI173294 |  |
| EPI173295 |  |
| EPI173298 |  |
| EPI173303 |  |
| EPI173314 |  |
| EPI173318 |  |
| EPI173439 |  |
| EPI173440 |  |
| EPI173441 |  |
| EPI173442 |  |
| EPI173443 |  |
| EPI173444 |  |
| EPI173445 |  |
| EPI173446 |  |
| EPI173715 |  |
| EPI173716 |  |
| EPI173717 |  |
| EPI173718 |  |
| EPI173794 |  |
| EPI174140 |  |
| EPI174156 |  |
| EPI175357 |  |
| EPI175358 |  |
| EPI175361 |  |
| EPI175389 |  |
| EPI175390 |  |
| EPI175391 |  |
| EPI175392 |  |
| EPI175393 |  |
| EPI175394 |  |
| EPI176111 |  |
| EPI176681 |  |
| EPI176793 |  |
| EPI176801 |  |
| EPI176993 |  |
| EPI177001 |  |
| EPI177009 |  |
| EPI177017 |  |
| EPI177025 |  |
| EPI177664 |  |
| EPI177672 |  |
| EPI177680 |  |
| EPI177688 |  |
| EPI178240 |  |
| EPI178325 |  |
| EPI179623 |  |
| EPI179655 |  |
| EPI179671 |  |
| EPI179679 |  |
| EPI179727 |  |
| EPI179743 |  |
| EPI179751 |  |
| EPI179791 |  |
| EPI179799 |  |
| EPI179807 |  |
| EPI179815 |  |
| EPI179823 |  |
| EPI179831 |  |
| EPI179839 |  |
| EPI179847 |  |
| EPI179855 |  |
| EPI181923 |  |
| EPI181931 |  |
| EPI182498 |  |
| EPI182664 |  |
| EPI182665 |  |
| EPI182666 |  |
| EPI182667 |  |
| EPI182668 |  |
| EPI182670 |  |
| EPI182671 |  |
| EPI182672 |  |
| EPI182673 |  |
| EPI182878 |  |
| EPI182986 |  |
| EPI182987 |  |
| EPI182988 |  |
| EPI182989 |  |
| EPI182990 |  |
| EPI182991 |  |
| EPI182992 |  |
| EPI183713 |  |
| EPI183714 |  |
| EPI183715 |  |
| EPI183716 |  |
| EPI183717 |  |
| EPI183718 |  |
| EPI183719 |  |
| EPI183720 |  |
| EPI183721 |  |
| EPI183722 |  |
| EPI183723 |  |
| EPI183724 |  |
| EPI183725 |  |
| EPI183726 |  |
| EPI183727 |  |
| EPI183728 |  |
| EPI184043 |  |
| EPI184044 |  |
| EPI184045 |  |
| EPI184046 |  |
| EPI184047 |  |
| EPI184048 |  |
| EPI184049 |  |
| EPI186321 |  |
| EPI186322 |  |
| EPI186323 |  |
| EPI186326 |  |
| EPI186327 |  |
| EPI186328 |  |
| EPI186330 |  |
| EPI186331 |  |
| EPI186332 |  |
| EPI186333 |  |
| EPI186336 |  |
| EPI186337 |  |
| EPI187294 |  |
| EPI187295 |  |
| EPI187296 |  |
| EPI187297 |  |
| EPI187298 |  |
| EPI187299 |  |
| EPI187300 |  |
| EPI187301 |  |
| EPI187302 |  |
| EPI187303 |  |
| EPI187304 |  |
| EPI187305 |  |
| EPI187306 |  |
| EPI187307 |  |
| EPI187308 |  |
| EPI189011 |  |
| EPI189453 |  |
| EPI189456 |  |
| EPI189459 |  |
| EPI189465 |  |
| EPI189468 |  |
| EPI189474 |  |
| EPI189477 |  |
| EPI189480 |  |
| EPI189483 |  |
| EPI189486 |  |
| EPI189489 |  |
| EPI189492 |  |
| EPI189495 |  |
| EPI189501 |  |
| EPI189504 |  |
| EPI189507 |  |
| EPI189510 |  |
| EPI189513 |  |
| EPI189516 |  |
| EPI189519 |  |
| EPI189522 |  |
| EPI189525 |  |
| EPI189527 |  |
| EPI189530 |  |
| EPI189533 |  |
| EPI189547 |  |
| EPI189569 |  |
| EPI189572 |  |
| EPI189575 |  |
| EPI189578 |  |
| EPI189627 |  |
| EPI189632 |  |
| EPI189640 |  |
| EPI189643 |  |
| EPI189646 |  |
| EPI189649 |  |
| EPI189658 |  |
| EPI189673 |  |
| EPI189676 |  |
| EPI189681 |  |
| EPI189714 |  |
| EPI189717 |  |
| EPI189720 |  |
| EPI189723 |  |
| EPI189726 |  |
| EPI189736 |  |
| EPI189739 |  |
| EPI189745 |  |
| EPI189747 |  |
| EPI189749 |  |
| EPI189752 |  |
| EPI189754 |  |
| EPI189756 |  |
| EPI189760 |  |
| EPI189766 |  |
| EPI189782 |  |
| EPI189785 |  |
| EPI189787 |  |
| EPI189793 |  |
| EPI189796 |  |
| EPI189799 |  |
| EPI189802 |  |
| EPI189805 |  |
| EPI189808 |  |
| EPI189811 |  |
| EPI189814 |  |
| EPI189817 |  |
| EPI189820 |  |
| EPI189823 |  |
| EPI189825 |  |
| EPI189828 |  |
| EPI189839 |  |
| EPI189842 |  |
| EPI189845 |  |
| EPI189848 |  |
| EPI189851 |  |
| EPI189856 |  |
| EPI189859 |  |
| EPI189862 |  |
| EPI189865 |  |
| EPI189868 |  |
| EPI189871 |  |
| EPI189874 |  |
| EPI189877 |  |
| EPI189880 |  |
| EPI189883 |  |
| EPI189891 |  |
| EPI189894 |  |
| EPI189899 |  |
| EPI189905 |  |
| EPI189908 |  |
| EPI189910 |  |
| EPI189912 |  |
| EPI189915 |  |
| EPI189918 |  |
| EPI189921 |  |
| EPI189924 |  |
| EPI189929 |  |
| EPI189932 |  |
| EPI189935 |  |
| EPI189938 |  |
| EPI189941 |  |
| EPI189946 |  |
| EPI189949 |  |
| EPI189954 |  |
| EPI189959 |  |
| EPI189962 |  |
| EPI189968 |  |
| EPI189971 |  |
| EPI189975 |  |
| EPI189978 |  |
| EPI189980 |  |
| EPI189983 |  |
| EPI189986 |  |
| EPI189988 |  |
| EPI189991 |  |
| EPI189994 |  |
| EPI189997 |  |
| EPI190000 |  |
| EPI190006 |  |
| EPI190009 |  |
| EPI190012 |  |
| EPI190015 |  |
| EPI190017 |  |
| EPI190019 |  |
| EPI190022 |  |
| EPI190025 |  |
| EPI190027 |  |
| EPI190030 |  |
| EPI190033 |  |
| EPI190036 |  |
| EPI190042 |  |
| EPI190045 |  |
| EPI190048 |  |
| EPI190051 |  |
| EPI190054 |  |
| EPI190060 |  |
| EPI190063 |  |
| EPI190066 |  |
| EPI190069 |  |
| EPI190075 |  |
| EPI190077 |  |
| EPI190079 |  |
| EPI190081 |  |
| EPI190084 |  |
| EPI190086 |  |
| EPI190089 |  |
| EPI190092 |  |
| EPI190101 |  |
| EPI190104 |  |
| EPI190107 |  |
| EPI190112 |  |
| EPI190138 |  |
| EPI190141 |  |
| EPI190143 |  |
| EPI190145 |  |
| EPI190169 |  |
| EPI190171 |  |
| EPI190172 |  |
| EPI190175 |  |
| EPI190176 |  |
| EPI190178 |  |
| EPI190179 |  |
| EPI190238 |  |
| EPI190278 |  |
| EPI190286 |  |
| EPI190294 |  |
| EPI191260 |  |
| EPI191261 |  |
| EPI191263 |  |
| EPI191264 |  |
| EPI191265 |  |
| EPI191266 |  |
| EPI191269 |  |
| EPI191271 |  |
| EPI191273 |  |
| EPI191279 |  |
| EPI191280 |  |
| EPI191281 |  |
| EPI191282 |  |
| EPI191283 |  |
| EPI191284 |  |
| EPI191285 |  |
| EPI191286 |  |
| EPI191287 |  |
| EPI191288 |  |
| EPI191289 |  |
| EPI191290 |  |
| EPI191295 |  |
| EPI191296 |  |
| EPI191297 |  |
| EPI191298 |  |
| EPI191299 |  |
| EPI191300 |  |
| EPI191301 |  |
| EPI191306 |  |
| EPI191307 |  |
| EPI191308 |  |
| EPI191309 |  |
| EPI191310 |  |
| EPI191311 |  |
| EPI191312 |  |
| EPI191313 |  |
| EPI191315 |  |
| EPI191316 |  |
| EPI191318 |  |
| EPI191319 |  |
| EPI191320 |  |
| EPI191321 |  |
| EPI191322 |  |
| EPI191324 |  |
| EPI191325 |  |
| EPI191326 |  |
| EPI191327 |  |
| EPI191329 |  |
| EPI191330 |  |
| EPI191331 |  |
| EPI191332 |  |
| EPI191333 |  |
| EPI191334 |  |
| EPI191335 |  |
| EPI191336 |  |
| EPI191337 |  |
| EPI191338 |  |
| EPI191339 |  |
| EPI191340 |  |
| EPI191341 |  |
| EPI191342 |  |
| EPI191343 |  |
| EPI191344 |  |
| EPI191345 |  |
| EPI191346 |  |
| EPI191347 |  |
| EPI191348 |  |
| EPI191349 |  |
| EPI191350 |  |
| EPI191351 |  |
| EPI191352 |  |
| EPI191353 |  |
| EPI191354 |  |
| EPI191356 |  |
| EPI191357 |  |
| EPI191358 |  |
| EPI191359 |  |
| EPI191360 |  |
| EPI191361 |  |
| EPI191362 |  |
| EPI191363 |  |
| EPI191364 |  |
| EPI191369 |  |
| EPI191370 |  |
| EPI191371 |  |
| EPI191372 |  |
| EPI191373 |  |
| EPI191374 |  |
| EPI191375 |  |
| EPI191376 |  |
| EPI191377 |  |
| EPI191378 |  |
| EPI191379 |  |
| EPI191380 |  |
| EPI191381 |  |
| EPI191382 |  |
| EPI191383 |  |
| EPI191384 |  |
| EPI191385 |  |
| EPI191386 |  |
| EPI191395 |  |
| EPI191396 |  |
| EPI191397 |  |
| EPI191398 |  |
| EPI191399 |  |
| EPI191400 |  |
| EPI191401 |  |
| EPI191402 |  |
| EPI191403 |  |
| EPI191405 |  |
| EPI191407 |  |
| EPI191408 |  |
| EPI191409 |  |
| EPI191410 |  |
| EPI191411 |  |
| EPI191413 |  |
| EPI191414 |  |
| EPI191419 |  |
| EPI191420 |  |
| EPI191421 |  |
| EPI191422 |  |
| EPI191423 |  |
| EPI191424 |  |
| EPI191425 |  |
| EPI191429 |  |
| EPI191430 |  |
| EPI191431 |  |
| EPI191432 |  |
| EPI191433 |  |
| EPI191434 |  |
| EPI191435 |  |
| EPI191436 |  |
| EPI191437 |  |
| EPI191439 |  |
| EPI192088 |  |
| EPI192120 |  |
| EPI192136 |  |
| EPI192152 |  |
| EPI192160 |  |
| EPI192168 |  |
| EPI192344 |  |
| EPI192368 |  |
| EPI192376 |  |
| EPI192472 |  |
| EPI192496 |  |
| EPI192997 |  |
| EPI193003 |  |
| EPI193012 |  |
| EPI193015 |  |
| EPI193020 |  |
| EPI193022 |  |
| EPI193028 |  |
| EPI193038 |  |
| EPI193847 |  |
| EPI193882 |  |
| EPI71977 |  |
| EPI72376 |  |
| EPI72490 |  |
| EPI72756 |  |
| EPI76326 |  |
| EPI76554 |  |
| EPI76573 |  |
| EPI93751 |  |
