# Supplementary material for: Selection for resistance to oseltamivir in seasonal and pandemic H1N1 influenza and widespread co-circulation of the lineages
Source: Int J Health Geogr. 2010 Feb 24;9:13. doi: 10.1186/1476-072X-9-13 (PMC2882220; doi:10.1186/1476-072X-9-13)
Supplement: Additional file 9 — Acknowledgements. Acknowledgements for researchers and institutions who submitted sequence data to GISAID and GenBank. [file 1476-072X-9-13-S9.DOC]

| **Individual Submitters** | **Sample Labs** |
| --- | --- |
| Carolyn Nicolson | Auckland Hospital |
| Catherine Smith | Childrens Hospital Westmead |
| Inmaculada Casas | Clinical Virology Unit |
| Jorge Osvaldo Fernandez | CSL Ltd |
| Juergen J Wenzel | Darwin Hospital |
| Kiku Mochizuki | IMVS |
| Makoto Ujike | Institute of Epidemiology and Infectious Diseases |
| Maria Brytting | Institute of Medical and Veterinary Science |
| Monica Galiano | Institute Pastuer NC |
| Naomi Komadina | Instituto de Salud Publica de Chile |
| Rebecca Garten | Inst. Of Environ. Science & Research |
| Takato Odagiri | Israel-NIC |
| Todd Davis | Laboratorio De Saude Publico |
| Valerie Caro | Laboratorio de Virus Respiratorio |
| Vicky Gregory | Monash Medical Centre |
| Yi-Mo Deng | National Institute for Communicable Disease |
| Yipu Lin | NIBSC |
| Yu Lan | Pathwest QE II Medical Centre |
| Yuki Furuse | Portugal-Lisbon |
|  | Queensland Health Scientific Services |
|  | Research Institute for Tropical Medicine |
|  | Singapore General Hospital |
|  | Swedish Institute for Infectious Disease Control |
|  | Victorian Infectious Diseases Reference Laboratory |
|  | Tohoku University Graduate School of Medicine |
|  | State Research Center of Virology and Biotechnology |
|  | NexBio, Inc. |
|  | Tehran University of Medical Sciences |
